# A conserved, noncanonical insert in FIS1 mediates TBC1D15 and DRP1 recruitment for mitochondrial fission

**DOI:** 10.1016/j.jbc.2023.105303

**Published:** 2023-09-28

**Authors:** Ugochukwu K. Ihenacho, Rafael Toro, Rana H. Mansour, R. Blake Hill

**Affiliations:** Department of Biochemistry, Medical College of Wisconsin, Milwaukee, Wisconsin, USA

**Keywords:** mitochondria, mitophagy, peroxisome, nuclear magnetic resonance (NMR), fission, tetratricopeptide repeat, repeat proteins, protein motif, organelle dynamic, dynamin

## Abstract

Mitochondrial fission protein 1 (FIS1) is conserved in all eukaryotes, yet its function in metazoans is thought divergent. Structure-based sequence alignments of FIS1 revealed a conserved, but noncanonical, three-residue insert in its first tetratricopeptide repeat (TPR) suggesting a conserved function. In vertebrates, this insert is serine (S45), lysine (K46), and tyrosine (Y47). To determine the biological role of the “SKY insert,” three variants were tested in HCT116 cells for altered mitochondrial morphology and recruitment of fission mechanoenzyme DRP1 and mitophagic adaptor TBC1D15. Similar to ectopically expressed wildtype FIS1, substitution of the SKY insert with alanine (AAA) fragmented mitochondria into perinuclear clumps associated with increased mitochondrial DRP1. In contrast, deletion variants (either ∆SKY or ∆SKYD49G) elongated mitochondrial networks with reduced mitochondrial recruitment of DRP1, despite DRP1 coimmunoprecipitates being highly enriched with ΔSKY variants. Ectopic wildtype FIS1 drove co-expressed YFP-TBC1D15 entirely from the cytoplasm to mitochondria as punctate structures concomitant with enhanced mitochondrial DRP1 recruitment. YFP-TBC1D15 co-expressed with the AAA variant further enhanced mitochondrial DRP1 recruitment, indicating a gain of function. In contrast, YFP-TBC1D15 co-expressed with deletion variants impaired mitochondrial DRP1 and YFP-TBC1D15 recruitment; however, mitochondrial fragmentation was restored. These phenotypes were not due to misfolding or poor expression of FIS1 variants, although ∆SKYD49G induced conformational heterogeneity that is lost upon deletion of the regulatory Fis1 arm, indicating SKY–arm interactions. Collectively, these results support a unifying model whereby FIS1 activity is effectively governed by intramolecular interactions between its regulatory arm and a noncanonical TPR insert that is conserved across eukaryotes.

In most eukaryotes, mitochondria exist as highly dynamic networks that balance frequent fission and fusion events to maintain the appropriate morphology necessary for organelle function and cellular homeostasis (1, 2, 3, 4). Early gene complementation screens in yeast revealed model genes, Dnm1, Mdv1/Caf4, and mitochondria fission 1 (Fis1), involved in mitochondrial fission (5, 6, 7, 8, 9, 10). These experiments led to a proposed rudimentary fission apparatus comprised of a resident outer membrane protein (Fis1p) acting in concert with an adaptor (Mdv1p/Caf4p) to recruit a GTPase mechanoenzyme (Dnm1p) from the cytoplasm to sites of scission (8, 9, 11, 12, 13, 14). However, only Fis1 and Dnm1 are present in all mitochondria-bearing species and Mdv1/Caf4 are fungal-specific with no known vertebrate orthologs identified to date. Moreover, increasing Fis1 expression potently induces Dnm1-dependent division of target organelles—mitochondria (15, 16, 17), peroxisomes, and plastids—regardless of species (18, 19, 20, 21, 22). These considerations suggest that proteins encoded by Fis1 and Dnm1 genes constitute the core fission machinery and that adaptors are unique from species to species. Supporting this idea are phylogenetic analyses that show high amino acid conservation across species (23, 24). Despite these considerations, the fission machinery in vertebrates is more complex as additional mitochondrial proteins like MFF and MID49/51 potently recruit the Dnm1 gene product, DRP1, in the absence of FIS1 (25, 26, 27, 28, 29). Groundbreaking work also identified that FIS1 recruits mitophagy adapters TBC1D15/17 to mitochondria, suggesting additional roles for FIS1 in vertebrates (30, 31, 32). In support, studies from simple to complex eukaryotes have described specific roles for FIS1 in peripheral mitochondrial fission during stress and/or development (33) with recent super resolution microscopy revealing that MFF recruits DRP1 for midbody, housekeeping fission, whereas FIS1 recruits DRP1 to peripheral sites for mitophagic removal (34). These findings suggest that FIS1 functional mechanisms have diverged in vertebrates despite the amino acid sequence conservation, thus raising the question of what governs FIS1 recruitment of TBC1D15/17 or DRP1.

Insights into FIS1 activity may be gained by consideration of its structure which has two domains: a C-terminal transmembrane domain that anchors it to membranes and a soluble helical domain that adopts a fold reminiscent of tetratricopeptide repeat (TPR) proteins (Fig. 1*A*) (35, 36). TPRs are 34 amino acid degenerate sequences that form a helix-turn-helix motif, occurring as three or more repeats to form superhelical arrays. This architecture creates a concave and convex face that mediates binding to multiple partners (37). To date, most TPRs seem to mediate binding *via* their concave face, access to which is often regulated by steric occlusion from flanking regions (38). FIS1 is an atypical TPR protein because it possesses two repeats, only one of which is canonical (35, 36). Furthermore, FIS1 exists in oligomeric heterocomplexes mediated by its TPRs, which may be autoinhibited by its N-terminal helix as deletion of this helix enhances FIS1 oligomerization and DRP1 recruitment (17, 23, 39, 40, 41). Adjacent to the N-terminal helix is a disordered region of FIS1, termed the FIS1 arm, that is required for its mitochondrial fission functions in both yeast and human cells (41, 42). Consistent with a key role for the N-terminal region are splice variants in mice and fruit flies that lack this region (43).

**Figure 1:**
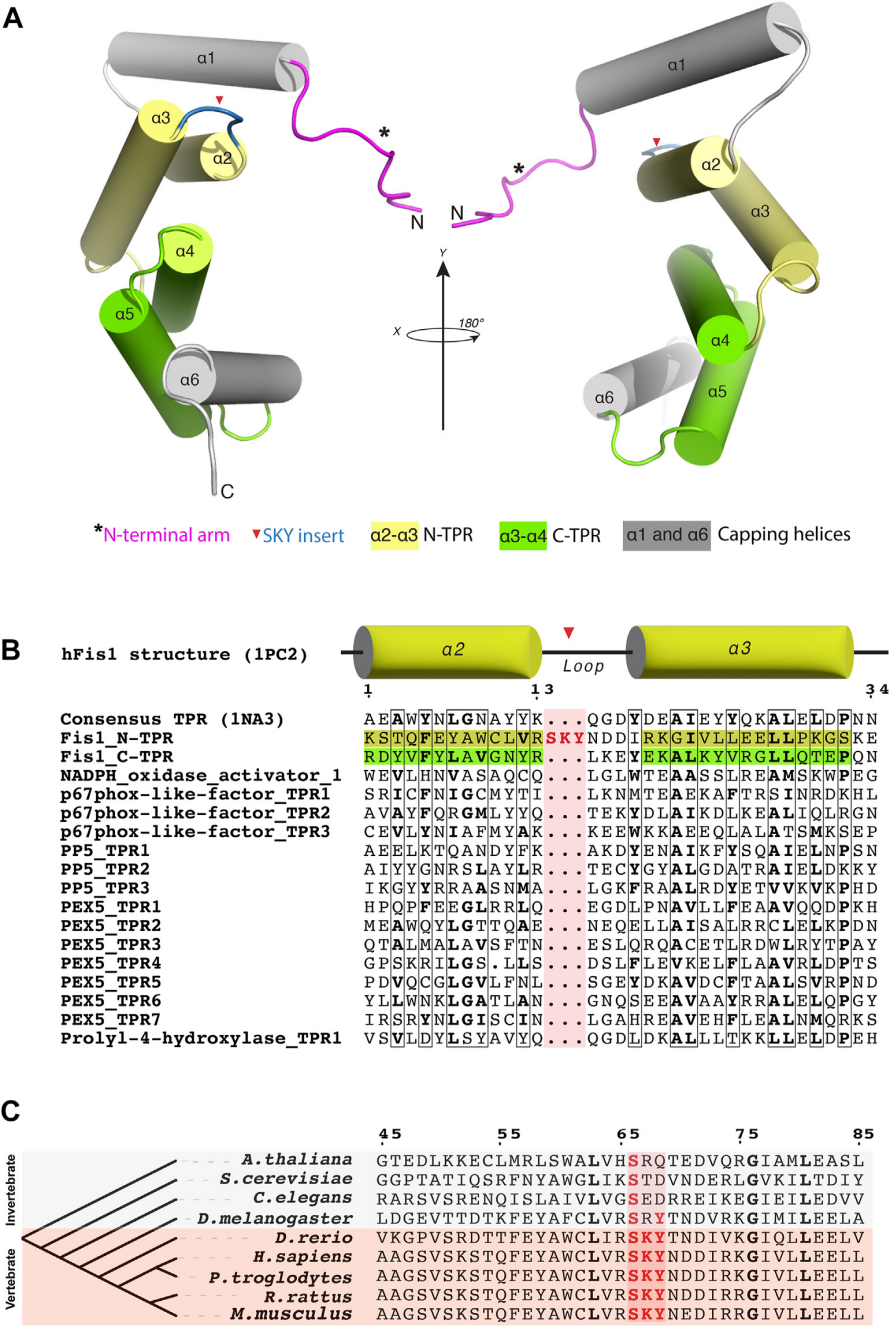
**Structure-based sequence alignments reveal a conserved three-residue insert in the N-terminal TPR of FIS1.***A*, solution structure of human FIS1 (PDB: 1PC2) depicting the N-terminal region called the “arm” (*red asterisk*), two tetratricopeptide repeats; the N-TPR in yellow (α-helices 2–3), and the C-TPR in green (α-helices 4–5) with flanking α-helices 1 and 6 in *gray*. The SKY insert (*red arrowhead*) is found in the turn of N-TPR in *blue* between α-helices 2 and 3. *B*, structure-based sequence alignments of human FIS1’s tetratricopeptide repeats and TPRs in the human proteome. The five-helix consensus TPR protein structure (PDB: 1NA03) was used as a template. Note that three-residues (Ser45, Lys46, Tyr47 in human) are inserted in the canonical TPR turn. *C*, the three-residue insert is conserved across FIS1 species and is always SKY in vertebrates. FIS1, fission protein 1.

In the current study, we searched for unifying mechanisms that could account for conservations of FIS1 functions and the observed differences between vertebrate and invertebrate species. Structure and phylogeny-based sequence alignments revealed a three-residue insert in the N-terminal TPR that is uniquely conserved as Ser-X-X in all species. Moreover, this insert is conserved as Ser-Lys-Tyr (SKY) in all vertebrates. Here, we report that the conserved SKY insert is not a stringent structural requirement for human FIS1 but is indispensable for its mitochondrial recruitment of TBC1D15 complexes that appear crucial to FIS1’s mitochondria division functions in vertebrates. Coimmunoprecipitation experiments suggests that ΔSKY variants drastically reduce TBC1D15 recruitment, although DRP1 recruitment is retained. Furthermore, we show that FIS1-induced fission of mitochondrial networks can be potently upregulated or downregulated by simply perturbing insert residues. Overall, our findings provide useful insights into elucidating unifying structural mechanisms that govern FIS1 activity and suggest differences between vertebrate and invertebrate FIS1 highlighted by insert residues.

## Results

### FIS1 has a conserved three-residue insert in the first TPR

We used structure-based sequence alignments to compare human proteins containing TPRs with FIS1 (Fig. 1*B*). Strikingly, these alignments revealed a noncanonical TPR feature in the first, but not the second TPR of FIS1: instead of the canonical 34 amino acids that define a TPR, FIS1’s first TPR (N-TPR) contains an additional stretch of three amino acids—serine, lysine, and tyrosine—inserted within the turn region of the canonical helix-turn-helix of a TPR (Fig. 1*B*). Curiously, a three-residue insert is present in all known FIS1 sequences and occurs as an invariant SKY in vertebrates (Fig. 1*C*). As the “SKY insert” is not required to specify the TPR fold, we infer that it is not conserved for structural purposes, but rather for FIS1 activity.

### Rational design and validation of SKY variants

**Figure 2:**
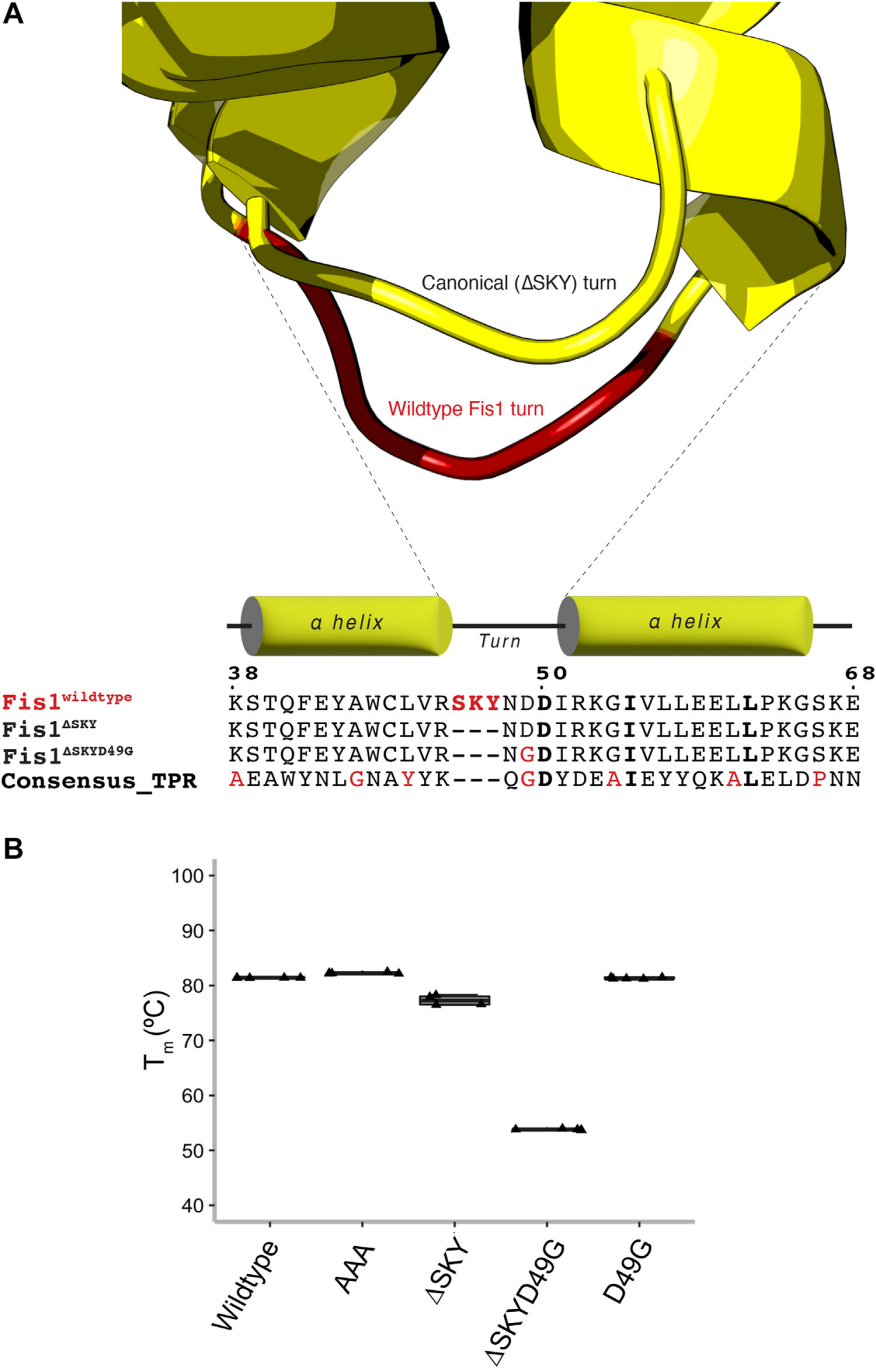

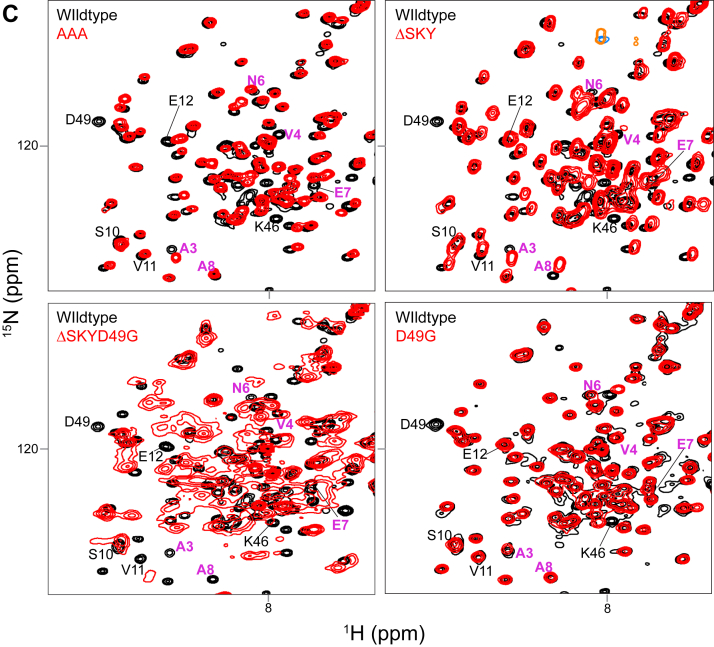
**Rational design and validation of a ΔSKY FIS1 variant.***A*, superposition of the N-TPR turn of FIS1 (PDB:1PC2) with a canonical TPR turn from the rationally designed, consensus TPR protein (PDB:1NA0). The ΔSKY construct removes the insert and ΔSKYD49G substitutes with a conserved Gly, see text for rationale. *B*, the midpoint of the thermal unfolding transition was determined by fitting light scattering data collected from 25 to 95 °C with the mean ± SD from 3 to 5 technical replicates shown as a *box-and-whisker plot*. *C*, ^1^H-^15^N HSQC spectral overlays of FIS1 WT (*black*) with indicated variants (*red*). Data were collected on 100 μM samples at 25 °C, pH 7.4 at 14.1 T. FIS1 arm cross peaks are indicated in *magenta*. See FigureS1 for full spectra overlays. FIS1, fission protein 1; HSQC, heteronuclear single quantum coherence; TPR, tetratricopeptide repeat.

To investigate the functional relevance of the SKY insert, we designed a FIS1 variant with a short canonical TPR turn lacking the insert. This was accomplished by analyzing the TPRs from a well-characterized consensus TPR sequence that adopts the canonical structure. This consensus TPR is an entirely non-native sequence designed from statistical thermodynamic analysis of TPR sequences and was shown to fold into the desired TPR structure, indicating the robustness of the design and TPR fold (44). Structural comparison of the FIS1 N-TPR with the consensus TPR from CTPR3 (1NA0.pdb) showed excellent alignment of the two helices (C_α_ RMSD = 1.1 Å) with only a slightly longer turn for FIS1 (Fig. 2*A*). This suggested that replacing the SKY insert with the turn from the TPR consensus sequence would not perturb the FIS1 fold. TPRs have a characteristic three-residue turn with φ, ψ backbone torsional angles that, according to Effimov’s convention (45), correspond to γ-α_L_-β of Ramachandran space with the central residue typically, but not always being a GLY that can readily adopt α_L_ values of φ, ψ space. Commonly the third position is a small, hydrophilic residue that adopts β space. Consistent with these principles, the consensus TPR turn is specified by the sequence Q-G-D, whereas the FIS1 turn is S-K-Y-N-D-D, with the SKY insert occurring before position 1. Deletion of the SKY insert leaves N-D-D to serve as the turn, which compares favorably to the consensus turn residues Q-G-D with the exception of the central GLY. Based on these considerations, we made four constructs by (i) substituting three Ala residues for SKY (AAA), (ii) deleting the SKY insert (ΔSKY) that retains the central Asp to give N-D-D, (iii) deleting the SKY insert that substitutes the central Asp (D49) with the canonical Gly (ΔSKYD49G) to give N-G-D, and (iv) a control that retained the SKY insert but replaced the succeeding Asp with Gly (D49G).

To assess the quality of our designs, we recombinantly expressed and purified the cytoplasmic domain of these proteins for biophysical characterization. Thermal stability was measured by monitoring the intrinsic fluorescence and light scattering with increasing temperature to determine the midpoint of the unfolding transition (T_m_). The WT cytoplasmic domain is quite thermally stable with a T_m_ of 81.8 ± 0.1 °C and neither alanine substitutions (AAA) nor the control construct (D49G) impacted thermal stability compared to WT (Fig. 2*B*). Deletion of the SKY insert (ΔSKY) modestly decreased the T_m_ to 71.5 ± 0.2 °C consistent with the assumption that these residues are dispensable for the TPR fold. However, the ΔSKYD49G construct dramatically decreased the T_m_ to 59.9 ± 0.2 °C. To understand this, we turned to two-dimensional NMR spectroscopy of these proteins uniformly labeled with ^15^N that allows for individual residue contributions to the overall protein fold. All constructs showed similar chemical shift dispersion to WT indicating well-folded proteins (Fig. 2*C*). However, ΔSKYD49G NMR data showed an increased broadening of resonances throughout the spectrum consistent with a significant degree of conformational heterogeneity. Moreover, cross peaks for N-terminal residues 1 to 8 corresponding to the “Fis1 arm” were not detected. To test the role of the FIS1 arm in this conformational heterogeneity, we created a ΔSKYD49G variant lacking the N-terminal arm (ΔNΔSKYD49G) and assessed its structure by thermal melt and NMR. Deletion of the FIS1 arm restored the T_m_ to a value similar to ΔSKY (73.6 ± 0.5 °C) and showed resonances largely similar to WT with little indication of conformational heterogeneity (Fig. S1). We interpret these data to indicate that the presence of the N-terminal arm was responsible for inducing conformational heterogeneity in ΔSKYD49G.

### The SKY insert is required for FIS1-induced changes in mitochondrial morphology

**Figure 3:**
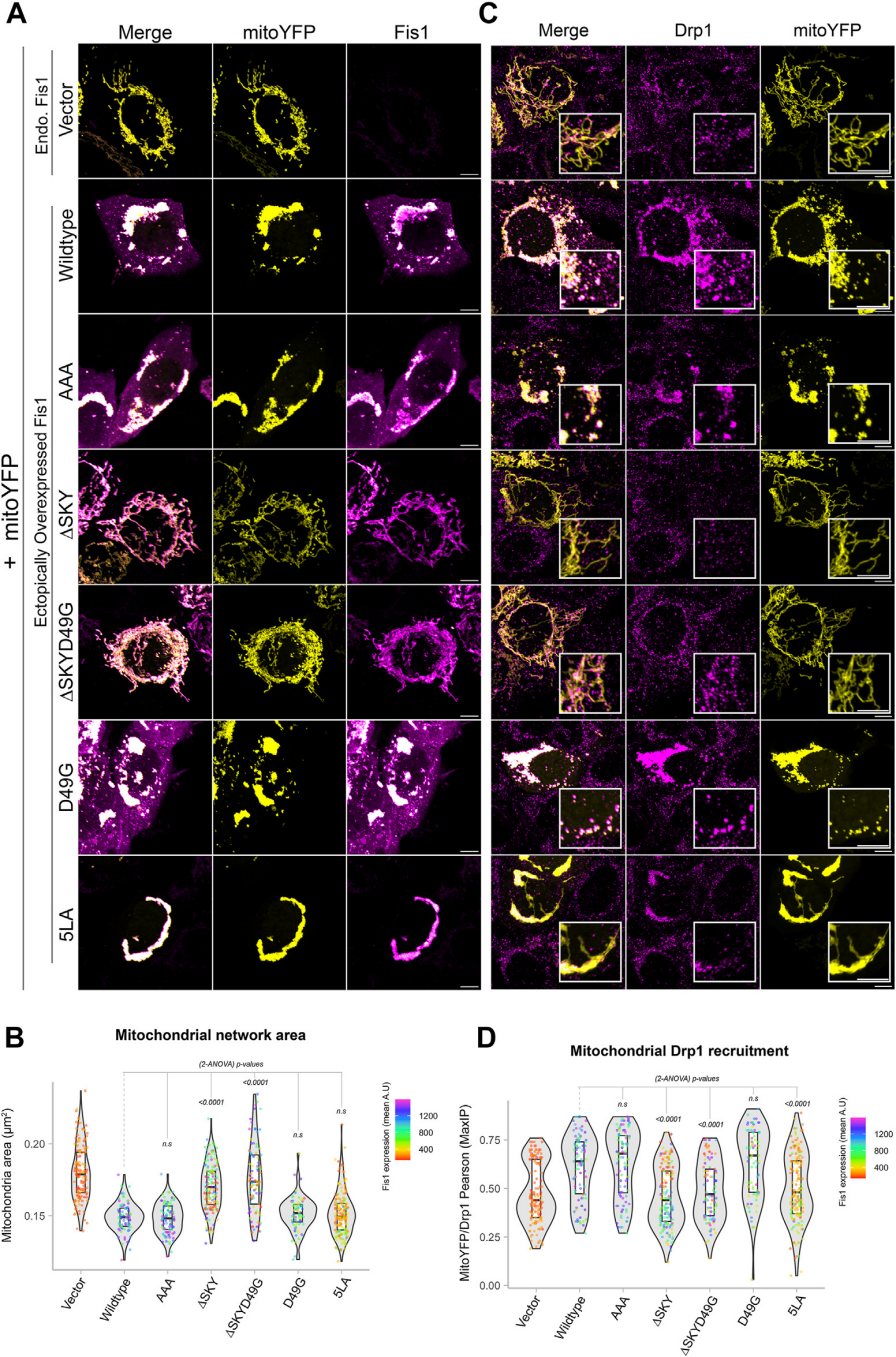

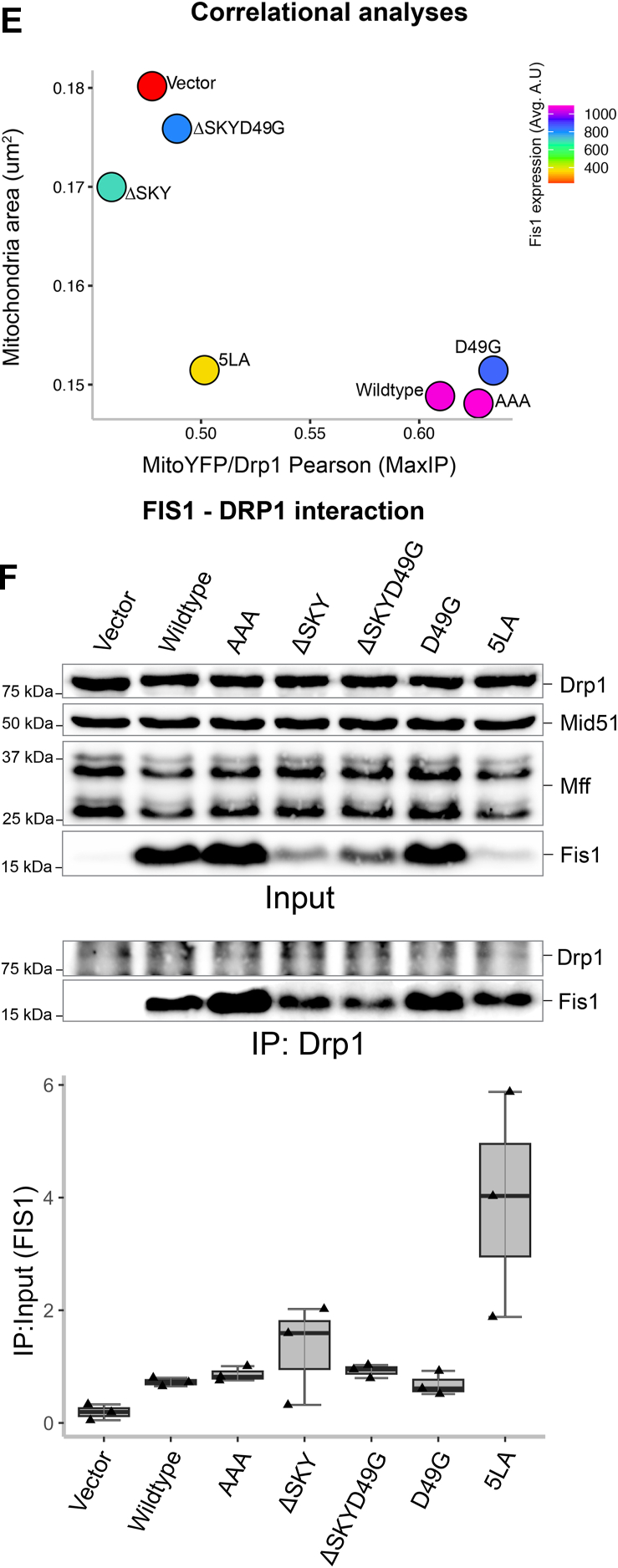
**The SKY insert is required for FIS1-induced changes in mitochondrial morphology.** HCT116 cells were transfected with mitoYFP and either pcDNA, pcDNA-FIS1 WT, or pcDNA-FIS1 variants as indicated, fixed, and immunostained sequentially for DRP1, followed by FIS1. *A*, representative confocal images showing merged anti-FIS1 (*magenta-hot*) and mitoYFP (*yellow*) from single channel images as indicated, the scale bar represents 10 μm. *B*, *violin plots* of average mitochondrial component area. *C*, representative confocal images showing merged anti-DRP1 (*magenta*) and mitoYFP (*yellow*) from single channel images as indicated. The scale bar represents 10 μm, (magnified scale bar represents 5 μm) with fluorescence intensities adjusted for clarity. *D*, *violin plot* of the colocalization between mitoYFP and DRP1 from single cell maximum intensity projections was measured using Pearson’s correlation coefficient. *E*, correlation plot to determine the relationship between mitochondrial network area and DRP1 recruitment. Each *point* in *B* and *D* represents a single cell and each *circle* in *E* represents the population means and are colored based on the FIS1 expression levels determined from mean fluorescence intensity per cell. Data represent three biological replicates with *p* values calculated from two-way ANOVA analyses followed by TUKEY honest significant differences (HSD). *F*, Western blots showing the input and DRP1 coimmunoprecipitated fractions harvested from PFA cross-linked cell lysates transfected with pcDNA or pCDNA-FIS1 and variants. The experiment was repeated three times, and FIS1–DRP1 complex formation is quantified on the *y*-axis as ratios of coimmunoprecipitate and input FIS1 signals. FIS1, fission protein 1; HCT, human colorectal carcinoma; PFA, paraformaldehyde.

To investigate the role of the SKY insert on cellular functions, we transiently expressed WT and FIS1 variants along with mitochondrially targeted YFP in human colorectal carcinoma (HCT116) cells. Ectopic overexpression of WT FIS1 induces uniform fragmentation and collapse of mitochondrial networks around the nucleus collectively resulting in perinuclear clumps confirming the findings by others (15, 16, 46, 47, 48) (Fig. 3*A*). We observed that protein expression levels between transfected FIS1 variants were not similar (Fig. S2*A*). Therefore, to rule out the possibility that morphological and/or functional changes between variants were simply a result of poor or inconsistent protein expression levels across the variants, we included only transfected cells that expressed moderate FIS1 levels for subsequent analyses (Fig. S2*B*). The changes in mitochondrial morphology were quantified by using MitoGraph (https://rafelski.com/susanne/MitoGraph) to determine the mitochondrial area, which showed a statistically significant decrease for WT compared to vector alone (Fig. 3*B*). As a control for the TPR domain, a commonly used FIS1 variant (5LA) that replaces five conserved TPR Leu residues with Ala was expressed (17, 30). As previously shown, the 5LA variant also caused mitochondrial clumping with a similar mitochondrial area to ectopic WT FIS1. Substituting AAA for the SKY insert closely phenocopied ectopic WT FIS1 with highly fragmented and clumped networks, also with similar mitochondrial areas. By contrast, removal of the SKY insert in both ΔSKY or ΔSKYD49G prevented fragmentation and network collapse with an increase of mitochondrial area that was statistically significant. This loss of function was not due to the D49G substitution as it showed mitochondrial morphology similar to WT expression.

The striking morphological changes induced by ectopic FIS1 involves mitochondrial recruitment of nonresident factors such as the highly conserved dynamin family GTPase, DRP1 (15, 16, 41). To evaluate this, we immunostained these cells for DRP1 and quantified colocalization with the MitoYFP signal (Fig. 3*C*). Mitochondrial recruitment of DRP1 is potently induced upon ectopic FIS1 overexpression consistent with earlier findings (15, 16, 41) (Fig. 3*D*). Therefore, we asked if mitochondrial DRP1 recruitment was significantly perturbed between WT and variant FIS1 overexpression in HCT116 cells. Consistent with an elongated mitochondrial network, mitochondrial DRP1 colocalization decreased by nearly 2-fold for both ΔSKY overexpressing cells. A similar decrease in mitochondrial DRP1 was observed for the known loss of function variant 5LA although expression of this variant induced mitochondrial clumping. In contrast, both AAA and D49G variants recruited DRP1 to mitochondria similar to WT FIS1. The reduction in mitochondrial area induced by FIS1 variants correlated reasonably well (R^2^ = 0.65) with their ability to recruit DRP1 with the notable exception of 5LA (Fig. 3*E*). We also noted less Drp1 signal in both the ΔSKY and 5LA expressing cells. To investigate if the observed differences in mitochondrial phenotypes were due to alterations in FIS1-DRP1 complex formation, we expressed variants in HCT116 cells, then harvested endogenous Drp1 complexes under cross-linking conditions was scored as the ratio of immunoprecipitated to input FIS1. Expectedly, we observed a nearly 4-fold increase in FIS1–DRP1 interaction when WT FIS1 (0.7 ± 0.1) was expressed compared to vector (0.2 ± 0.1) alone. Compared to WT FIS1, AAA had slightly higher ratios (0.9 ± 0.1), while D49G had the least ratio of all variants (0.7 ± 0.3). Unexpectedly, we observed high ratios for loss of function variants ΔSKY, ΔSKYD49G, and 5LA (1.3 ± 0.9, 0.9 ± 0.2, and 3.9 ± 1.9, respectively), suggesting that compared to WT FIS1 expression, there is an increase in FIS1–DRP1 interaction (Fig. 3*G*). This unexpected trend suggests that loss of function variants (ΔSKY, ΔSKYD49G, and 5LA) can still recruit DRP1 in the absence of fission. The abnormally high ratios observed in ΔSKY and 5LA (1.31 ± 0.9 and 3.92 ± 1.9) are consistent with the modest expression of these variants that was restricted to the mitochondria and is in contrast to ectopic WT FIS1 that was less restricted and also highly expressed in the cytosol (Fig. 3*A*). Taken together, our results indicate that deleting the SKY insert leads to large perturbations in FIS1–DRP1 complex formation that appear to impair mitochondrial fission.

### The FIS1 SKY insert is required for effective mitochondrial recruitment of TBC1D15

**Figure 4:**
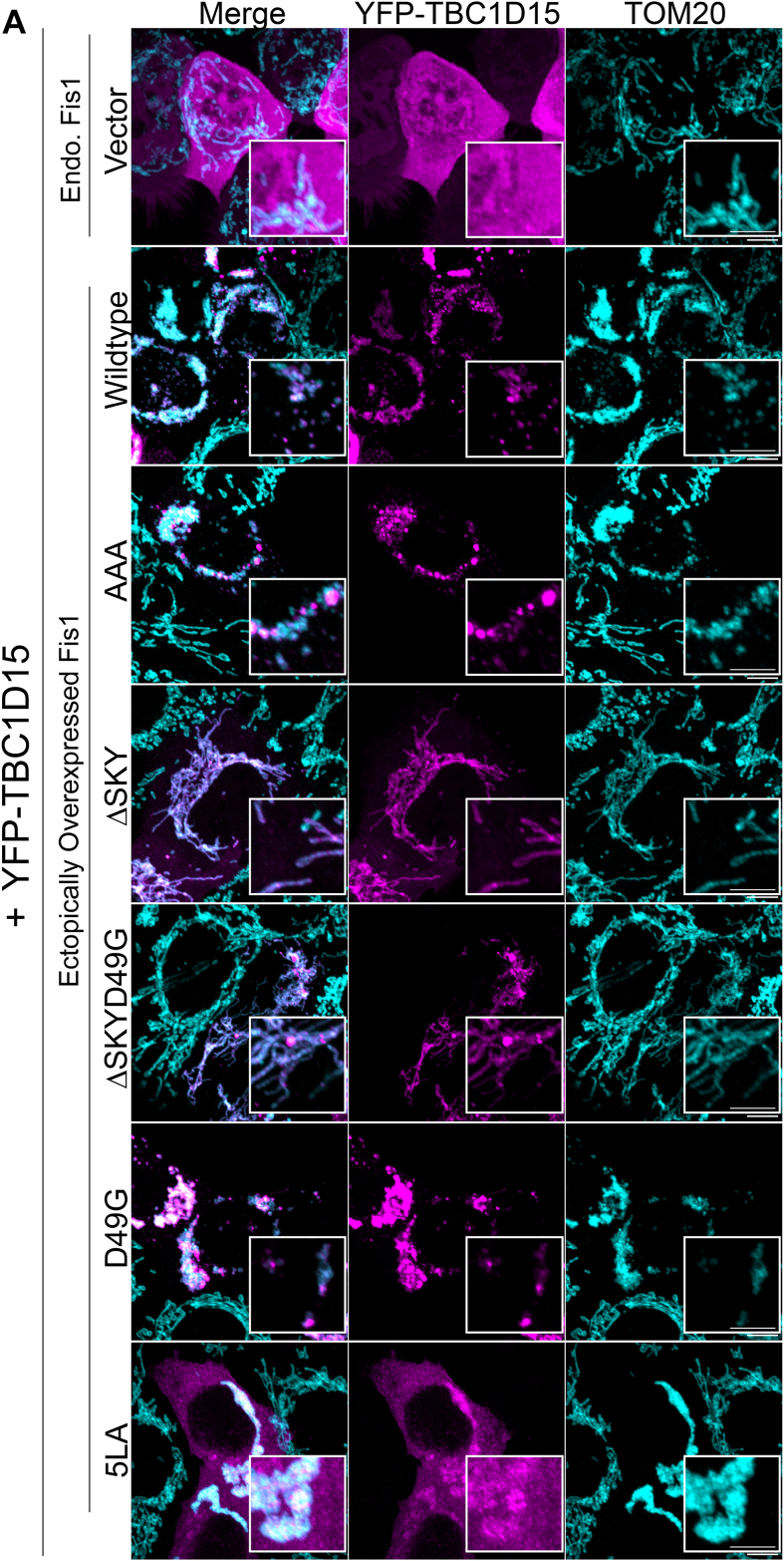

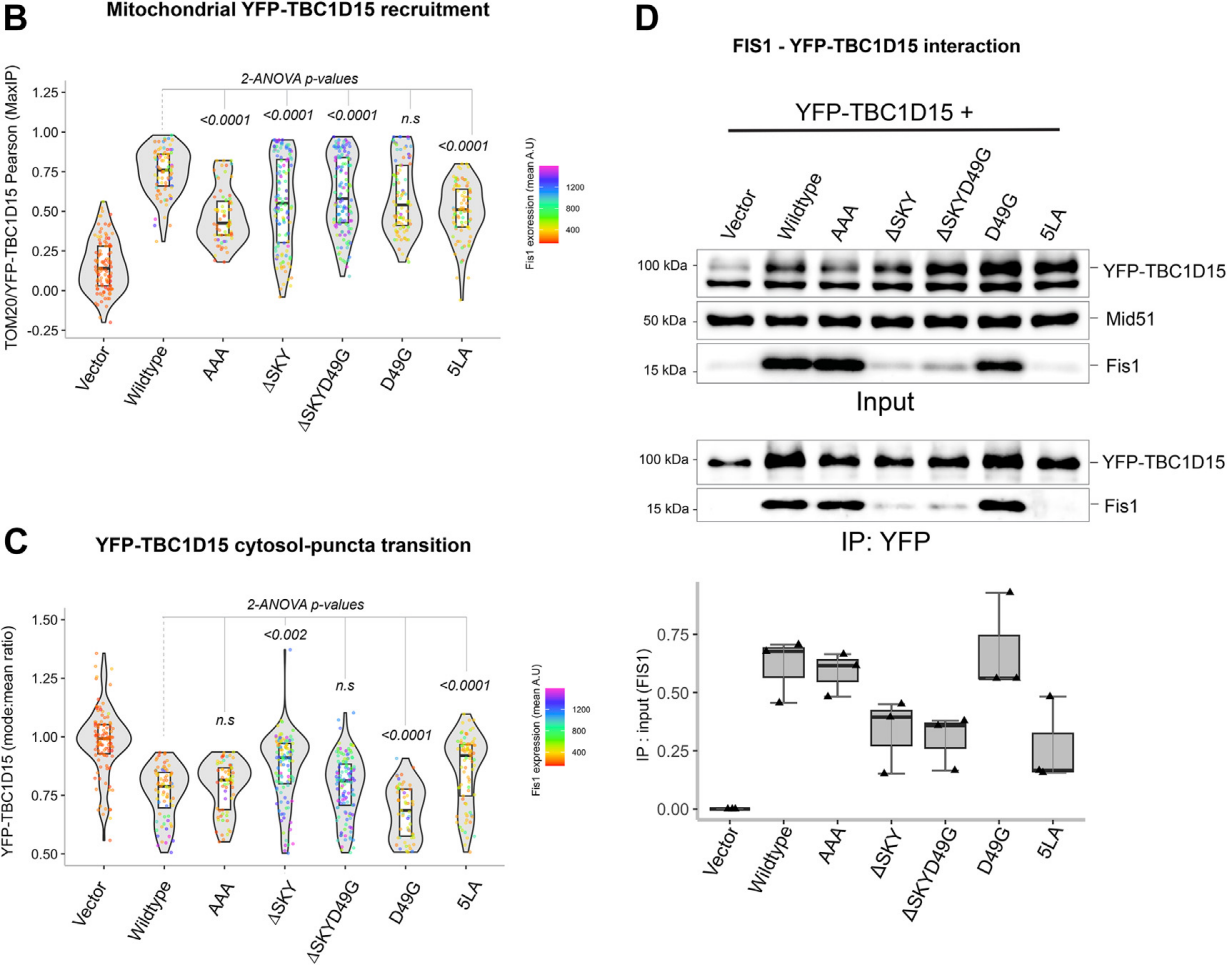
**The FIS1 SKY insert is required for effective mitochondrial recruitment of TBC1D15.** Analyses of HCT116 cells co-overexpressing FIS1 and YFP-TBC1D15. *A*, *from right to left*, representative confocal images of TOM20 (*cyan*) immunostained cells ectopically expressing YFP-TBC1D15 (*magenta*), and merges of both channels (merged). The scale bar represents 10 μm (magnified inset scale bar represents 5 μm). *B*, *violin plots* of YFP-TBC1D15 puncta assembly assessed by differences in mode and mean fluorescence intensity values. The *top panel* shows the mean YFP-TBC1D15 signal intensities, and the *bottom panel* shows ratios of modal and mean signal intensities. Ratio values close to 1 are indicative of no puncta assembly. *C*, *violin plot* of the colocalization between TOM20 and YFP-TBC1D15 from single cell maximum intensity projections was measured using Pearson’s correlation coefficient. Each data point is colored based on the FIS1 expression levels determined from the mean fluorescence intensity per cell. Data represent three biological replicates with *p* values calculated from two-way ANOVA analyses, followed by TUKEY honest significant differences (HSD). *D*, Western blots showing the input and YFP coimmunoprecipitated fractions harvested from PFA cross-linked cell lysates. The experiment was repeated three times, and the FIS1–YFP-TBC1D15 interaction is quantified on the *y*-axis as ratios of coimmunoprecipitate and input FIS1 signals. FIS1, fission protein 1; PFA, paraformaldehyde.

The FIS1 TPR domain is exposed to the cytoplasm, where it also recruits other binding partners to help govern mitochondrial network morphology. One such class of proteins are the cytoplasmic TBC1 effectors important to many cellular functions, including serving as GTPase-activating proteins for Rab family proteins. One TBC1 protein recruited by FIS1 is TBC1D15, and we next explored if the SKY variants impacted TBC1D15 recruitment. For this, the FIS1 constructs were cotransfected with YFP-TBC1D15, and mitochondrial networks were visualized by immunofluorescence of the mitochondrial outer membrane marker TOM20 (Fig. 4*A*). Mitochondrial recruitment of YFP-TBC1D15 was evaluated by measuring colocalization between YFP and immunostained TOM20 (Fig. 4*B*). Without FIS1 overexpression, the TBC1D15 signal is predominantly cytoplasmic and does not concentrate on mitochondrial networks, consistent with endogenous FIS1 levels in HCT116 cells being quite low (Fig. 4*B*). By contrast, WT FIS1 expression triggers a robust transition of cytosolic TBC1D15 pools onto mitochondrial sites as discrete foci or puncta, which was concomitant with FIS1-induced mitochondrial fragmentation and perinuclear clumping. For FIS1 variants, coexpression of YFP-TBC1D15 impaired the YFP-TBC1D15 cytoplasm-to-puncta transition. To quantify this transition, the mean and mode values of cellular YFP-TBC1D15 signal were measured and reported as mean:mode ratios (Fig. 4*C*). For vector alone, the mean and mode are essentially equivalent, reflecting the even distribution. For WT FIS1 expression, the mean:mode ratio decreases by 40%, reflecting a decrease of uniform, cytoplasmic YFP-TBC1D15, and the formation of TBC1D15 punctate structures that reside on mitochondrial surfaces (Fig. 4*C*). As expected, the 5LA variant impaired both mitochondrial recruitment and formation of TBC1D15 puncta with similar mean:mode ratios to vector alone (Fig. 4, *B* and *C*). In the case of AAA, puncta formation appeared to be dysregulated because compared to WT, YFP-TBC1D15 mitochondrial recruitment was reduced by half (Fig. 4*B*), and puncta formation appeared unperturbed (Fig. 4*C*). Although, punctate structures in AAA were noticeably larger but fewer than WT as indicated by the increase in mean:mode values. Both ΔSKY constructs similarly impaired mitochondrial recruitment, and puncta formation was almost completely abolished by ΔSKY (Fig. 4, *B* and *C*). ΔSKYD49G caused the formation of fewer punctate structures, a phenotype that is between ΔSKY and D49G, the latter of which appeared to be more effective at driving puncta formation than WT (Fig. 4*C*). To determine if these observed changes were indeed due to disruptions in Fis1–TBC1D15 interactions, we isolated TBC1D15 complexes from cells coexpressing FIS1 and YFP-TBC1D15 by coimmunoprecipitation and then probed for FIS1. In line with previous reports, we observed that TBC1D15–Fis1 complex formation is almost nonexistent at endogenous FIS1 levels (Fig. 4*D*). In contrast, complex formation is robust when WT FIS1 or D49G is ectopically expressed with YFP-TBC1D15. In line with previous reports, FIS1–TBC1D15 complexes were significantly disrupted by 5LA. Similarly, complex formation was impaired by AAA and even more so for both ΔSKY constructs (Fig. 4*D*). We, therefore, conclude that the FIS1 SKY insert is required for proper TBC1D15 recruitment.

### FIS1-mediated fission is potentiated by TBC1D15; loss of DRP1 recruitment is partially rescued by TBC1D15 overexpression

**Figure 5:**
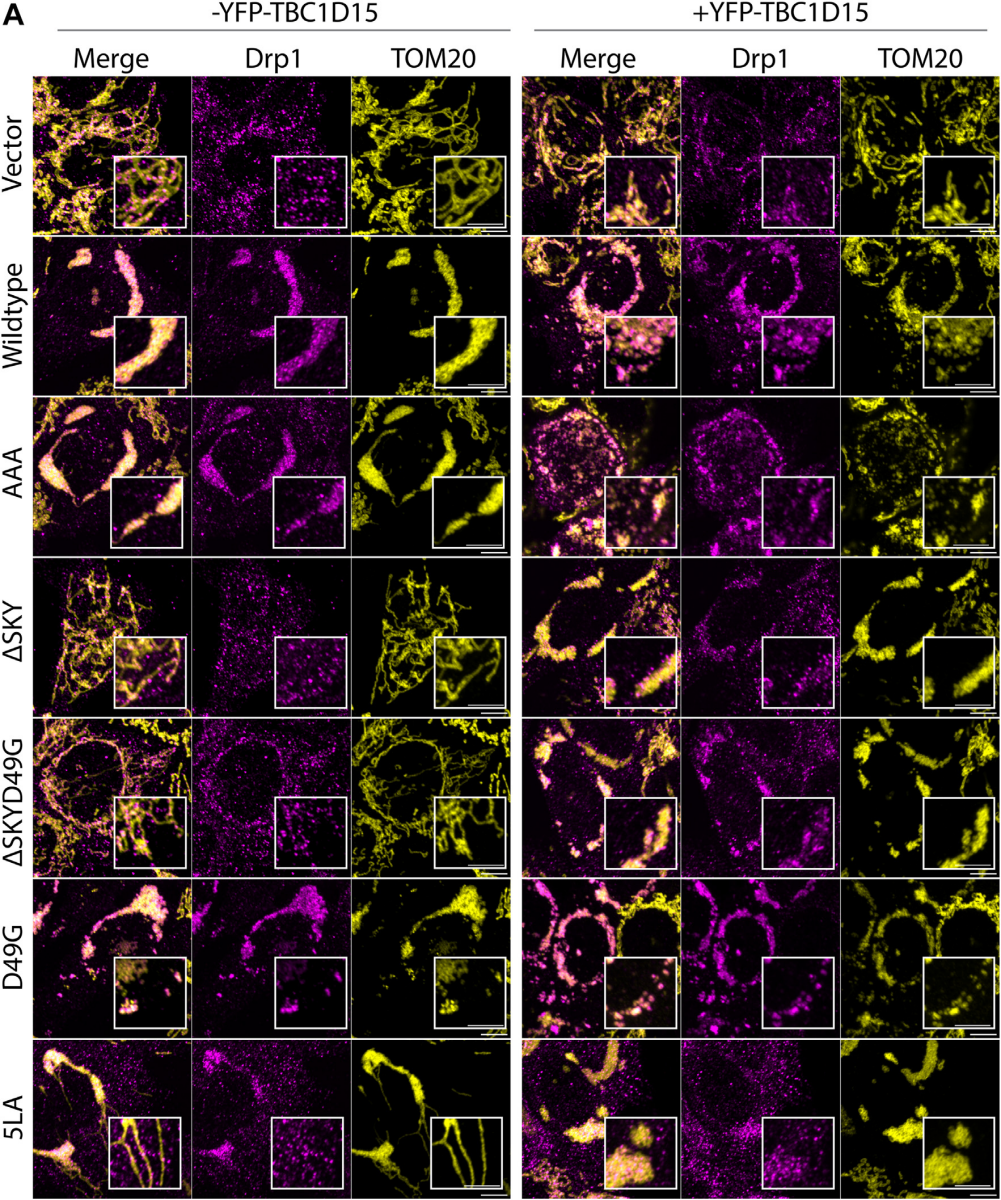

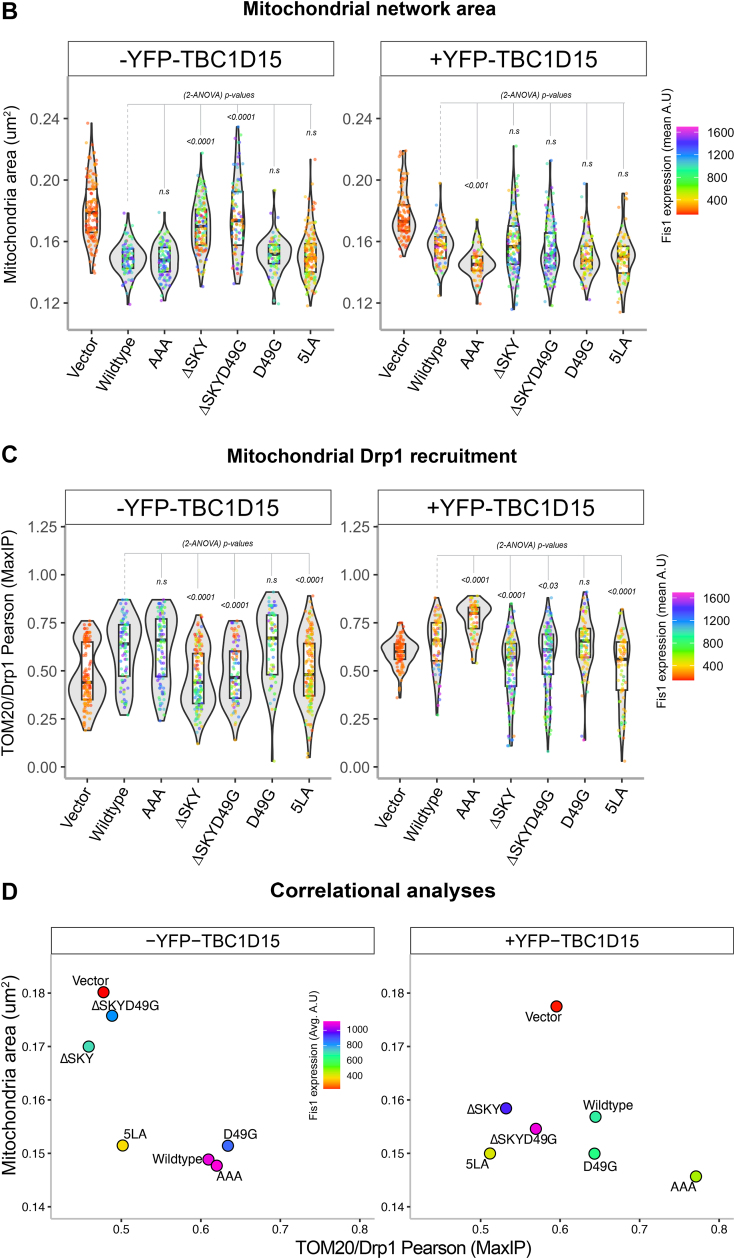
**Fis1 ∆SKY variants loss of function is rescued by TBC1D15 expression.** The impact of YFP-TBC1D15 expression on mitochondrial morphology and DRP1 localization was determined from experiments shown in Figures 3 and 4; HCT116 cells co-overexpressing FIS1 with either mitoYFP (-YFP-TBC1D15, from Fig. 3 experiments), or YFP-TBC1D15 (+YFP-TBC1D15, from Fig. 4 experiments) were analyzed for mitochondrial morphology and DRP1 localization. *A*, representative confocal images showing merged anti-DRP1 (*magenta*) and anti-TOM20 (*yellow*) from single channel images before (*left panel*) and after (*right panel*) transfection with YFP-TBC1D15. Note for ΔSKYD49G, the Figure 3*C* image are reused in *A*. The scale bar represents 10 μm (magnified inset scale bar represents 5 μm) with fluorescence intensities adjusted for clarity. *B*, *violin plots* of average mitochondrial component area in absence (*left panel*, from Fig. 3*B*) and presence (*right panel*) of YFP-TBC1D15 coexpression. *C*, *violin plot* of the colocalization between TOM20 and DRP1 from single cell maximum intensity projections was measured using Pearson’s correlation coefficient area in absence (*left panel*, from Fig. 3*D*) and presence (*right panel*) of YFP-TBC1D15 coexpression. *D*, *correlation plot* to determine the relationship between mitochondrial component area and mitochondrial DRP1 in absence (*left panel*, from Fig. 3*E*) and presence (*right panel*) of YFP-TBC1D15 coexpression. Each *point* in *B* and *C* represents a single cell and each *circle* in *D* represents the population means and are colored based on the FIS1 expression levels determined from mean fluorescence intensity per cell. Data represent three biological replicates with *p* values calculated from two-way ANOVA analyses, followed by TUKEY honest significant differences (HSD). FIS1, fission protein 1; HCT, human colorectal carcinoma.

We next asked if coexpression of TBC1D15 with FIS1 variants impacted mitochondrial morphology and DRP1 recruitment. To ensure that functional analyses were not biased by differential protein expression levels, we again gated for only cells that moderately expressed FIS1 (Fig. S3, *A* and *B*). In the absence of exogenous TBC1D15, the AAA variant drove a similar clumped morphology to WT (Fig. 5*A*, left panel), which was quantified again by using MitoGraph to measure the mitochondrial area (Fig. 5*B*, left panel). Surprisingly, compared to WT, coexpression of AAA with YFP-TBC1D15 caused mitochondrial clumps to resolve into much smaller clumps, indicated by a 10% decrease in mitochondria (Fig. 5, *A* and *B*, right panels). Coexpression of either ΔSKY constructs with YFP-TBC1D15 reversed the elongated mitochondrial morphology of these variants as indicated by similar mitochondrial areas to WT (Fig. 5, *A* and *B*). Coexpression of D49G with YFP-TBC1D15 led to decreased mitochondrial area consistent with its increased TBC1 recruitment. By contrast, coexpression of the 5LA variant did not show increased mitochondrial fragmentation with YFP-TBC1D15, consistent with 5LA’s defective ability to support TBC1 recruitment onto mitochondrial sites. These results indicate that insert perturbations modulate FIS1 activity through TBC1D15 recruitment, since ΔSKY loss of function is rescued by TBC1D15 expression, supporting an integral role for TBC1D15 in FIS1-driven changes in mitochondrial morphology.

To determine whether this rescue depended on DRP1, we immunostained for endogenous DRP1 in these experiments and assessed mitochondrial colocalization (Fig. 5, *A* and *C*). Across all conditions, expression of TBC1D15 increased mitochondrial localization of DRP1. This is most notable for vector-transformed cells with endogenous FIS1 expression, as they showed a bimodal distribution of DRP1 recruitment in the violin plot and a reversal of the DRP1 poor recruitment phenotype upon TBC1D15 expression (Fig. 5*C*). In the presence of TBC1D15, the AAA variant increased DRP1 recruitment by over 20% compared to WT consistent with its more pronounced effect on mitochondrial morphology with decreased mitochondrial area. D49G showed a similar effect, although not statistically significant with respect to DRP1 localization. For the ΔSKY variants, we observed a bimodal distribution of DRP1 localization in the absence of TBC1D15 coexpression, which was similar to vector alone. This bimodal distribution was also eliminated upon TBC1D15 coexpression, although these ΔSKY variants still had impaired DRP1 localization compared to WT (Fig. 5, *A* and *C*). Similar results were found for the 5LA variant. These data indicate that the expression of TBC1D15 potentiates mitochondrial DRP1 recruitment and partially rescues the fission defect in FIS1 ΔSKY variants. For example, ΔSKY variants reduced mitochondrial area while concomitantly increasing DRP1 colocalization (Fig. 5*D*). Interestingly, these correlational analyses revealed that ectopic TBC1D15 unmasks significant functional differences between WT FIS1 and the AAA variants that are otherwise obscured, suggesting that FIS1’s fission activity is dependent on TBC1D15 (Fig. 5*D*).

## Discussion

Here, we report that mitochondrial fragmentation and perinuclear clumping typical of WT FIS1 overexpression were abolished upon deletion of the SKY insert, which we show is a noncanonical yet highly conserved insert into the N-terminal TPR of FIS1 (Fig. 1). In this manuscript, the observed reduction in mitochondrial area and perinuclear clumping of networks are regarded as “endpoint” effects of ectopic FIS1 expression (Figure 3, Figure 4, Figure 5). Ectopically expressed WT FIS1 triggers unopposed mitochondrial fission in a Drp1-dependent manner. However, Fis1 overexpression without commensurate recruitment of fission effectors—such as TBC1D15 and DRP1—also leads to perinuclear clumping, as was the case with the known variant (5LA), which apparently lacks recruitment activity but still induces mitochondrial clumps. Both ΔSKY variants reduce DRP1 recruitment to mitochondria, supporting a role for FIS1 in DRP1-mediated fission. Both ΔSKY variants reduced exogenous TBC1D15 recruitment to mitochondria and could not support TBC1D15 assembly into punctate structures, indicating that the SKY insert also supports functionally important interactions with TBC1D15. Ectopic TBC1D15 expression increased mitochondrial DRP1 localization in all conditions regardless of which FIS1 construct was coexpressed and likely explains the partial rescue of mitochondrial morphology upon coexpression with ΔSKY variants. An important role for the SKY insert in FIS1 activity is also supported by slight gains of function activities found for AAA and D49G variants in the presence of ectopic TBC1D15. Interestingly, ectopic TBC1D15 also resolved mitochondrial clumps, presumably by potentiating mitochondrial DRP1 recruitment and fission. Thus, our results indicate that impairments to the mitochondrial fission machinery can have dominant effects—phenotypically in the form of mitochondrial clumping—that are mitigated by augmenting mitochondrial fission.

**Figure 6:**
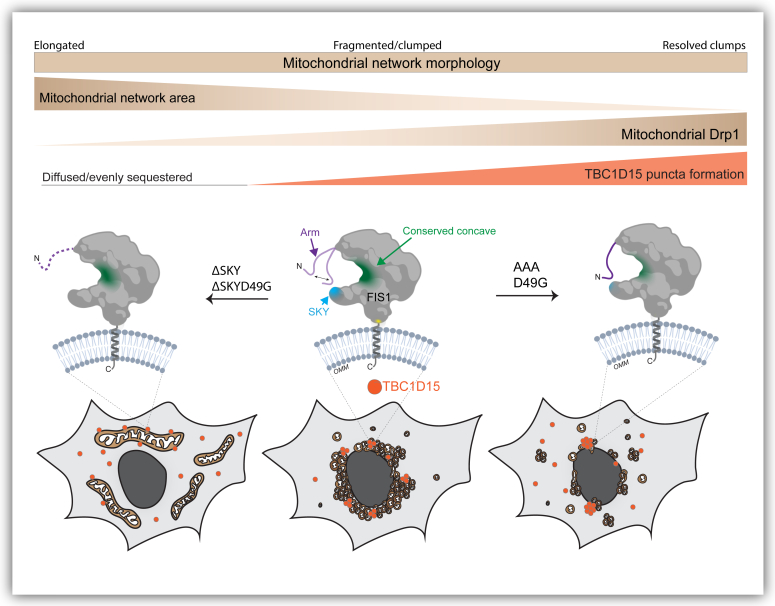
**The conserved SKY insert helps to govern recruitment of DRP1 and TBC1D15 in fission.** The three-residue insert in FIS1 is conserved across species for DRP1 and species-specific adaptor recruitment *via* conserved interactions with the FIS1 arm. FIS1, fission protein 1.

Previously, we reported that deletion of the first eight residues of FIS1, termed the FIS1 arm, impaired DRP1 localization, and mitochondrial fission (41). Here, we find a similar effect in HCT116 cells upon deletion of the SKY insert, but not substitution of these residues with AAA. Both arm and SKY deletions potently impair FIS1 activity and mitochondrial DRP1 recruitment. Interestingly, we also noted that both arm and SKY deletions prevented ectopic TBC1D15 puncta formation; instead, TBC1D15 was uniformly sequestered on mitochondrial networks indicating that the FIS1 arm does not directly mediate binding, but likely regulates other interactions necessary for TBC1D15 puncta formation (41) (Fig. 4). These observations are likely connected: molecular dynamics simulations show intramolecular, bifurcated hydrogen-bonding between the carboxamide of Asn6 in the FIS1 arm, and the backbone atoms of the SKY insert are possible (41). Such interactions would be expected to be supported by AAA and D49G, but not ΔSKY variants. NMR chemical shift changes in arm residues upon deletion of SKY also support the possibility of arm–SKY intramolecular interactions (Fig. 2). Moreover, the NMR data for ΔSKYD49G shows conformational heterogeneity that is relieved upon deletion of the FIS1 arm (Fig. S1), indicating that the arm is responsible for this heterogeneity; it is likely indiscriminately sampling non-native interactions with the TPR core in the absence of the SKY insert. The thermal unfolding data are also consistent with this interpretation as arm deletion restores the T_m_ to 73.6 ± 0.5 (not shown). Thus, multiple lines of evidence support that FIS1 activity requires intramolecular arm–SKY interactions that might govern the recruitment and assembly of effector proteins like TBC1D15 and DRP1 (Fig. 6).

Ectopic expression of YFP-TBC1D15 increases DRP1 localization under all conditions tested, including endogenous conditions, which partially rescues the mitochondrial fission defects caused by ectopic expression of FIS1 ΔSKY variants (Fig. 5). These data indicate that TBC1D15 can drive mitochondrial fission *via* endogenous FIS1 and/or mechanisms that are FIS1-independent, since TBC1D15 also physically interacts with DRP1 (49). In this sense, these data are highly reminiscent of the Fis1p-Mdv1p-Dnm1p apparatus in yeast, where deletion of the FIS1 arm can be rescued upon Mdv1p overexpression, which also has known interactions with Dnm1p, the yeast DRP1 ortholog (8). Thus, it is reasonable to consider that TBC1D15 may be a functional Mdv1p ortholog in vertebrates. However, Mdv1p and TBC1D15 only share 21% sequence identity and share no discernible structural homology based on AlphaFold predictions except for disordered regions, supporting the idea that FIS1 likely has species-specific adaptors.

Ectopic expression of YFP-TBC1D15 significantly reduced mitochondrial clumps caused by FIS1 overexpression, as witnessed by reduced mitochondrial area (Fig. 5). One plausible explanation is that TBC1D15 resolves mitochondrial clumps by increasing effective fission rates, since it can further facilitate the mitochondrial recruitment of DRP1. Another possibility is that TBC1D15 induces a structural conformation necessary for the activation of FIS1 activity. YFP-TBC1D15 expression also stabilized FIS1 (Fig. S3*E*), and thus links TBC1D15 to both Fis1 activity and turnover (30). We note that although AAA and WT FIS1 are functionally similar, AAA expression was significantly higher than WT, indicating that FIS1 activity and turnover are disrupted by the AAA mutation (Figs. S2*A* and S3*E*). Interestingly, in the presence of YFP-TBC1D15, the AAA FIS1 variant shows a significant gain of function phenotype compared to WT (Fig. 5). FIS1 turnover is likely regulated by posttranslational modifications, with ubiquitination playing a central role (50, 51, 52, 53). For instance, ubiquitin-mediated FIS1 turnover in lipogenic cells is inhibited by the deacetylation of unknown lysine residues that may include K46 of the SKY insert (51).

TBC1D15 is reported to have oncogenic, lysosomal, and mitophagic functions (31, 54, 55, 56, 57, 58) presumably by interacting with p53-Numb, FIS1-DRP1, and Rab7A (30, 49, 54, 55). Interestingly, FIS1’s mitophagic functions appear to be closely linked to TBC1D15-dependent tethering of Rab7A+ subcellular compartments to mitochondrial sites (31, 57). Functional links between FIS1 and TBC1D15 are demonstrated by gene knockout studies, showing synergistic increases in mitochondrial elongation upon ablating both FIS1 and TBC1D15 (30). As such, the TBC1D15 protein may function as a limiting factor for autophagolysosomal fusion mediated by Rab7A during mitophagy (31, 32). Interestingly, genetic ablation of FIS1 or TBC1D15 led to the formation of large LC3B structures that are indicative of impaired autophagy, providing further evidence of functional links between FIS1 and TBC1D15 (31). Our work extends these observations by showing a new role for TBC1D15 in facilitating FIS1-mediated DRP1 recruitment. TBC1D15 is actively degraded during nutrient starvation (54), a stressor that also triggers mitochondrial elongation in mammalian cells (59), which in light of these results, may be a consequence of reduced FIS1-mediated fission.

## Experimental procedures

### Structural and phylogenetic sequence alignments

We searched PROSITE for human proteins containing TPRs (60). Putative TPR sequences alone from these proteins were then manually compiled as a FASTA formatted file and aligned on PROMALS3D using the synthetically designed TPR structure (PDB:1NA0) as a template (61). The alignment file generated by PROMALS3D was used to render the alignment figure on ESPript3.0 (62), and annotations were added to the final figure using Adobe Illustrator.

### Protein expression and purification

The soluble domains of FIS1 and variants were recombinantly expressed as SUMO protease cleavable 6xHis-smt3 fusion constructs in *Escherichia coli* BL21DE3(pRep4) cells as previously described (63). Postcleavage of the 6xHis-smt3 tag with recombinant SUMO, FIS1 constructs were purified to homogeneity using nickel affinity and size-exclusion chromatography as described previously (63). Subsequently, samples were buffer exchanged into the final experimental buffer (100 mM Hepes pH 7.4, 200 mM NaCl, 1 mM DTT, 0.02% (w/v) sodium azide) for storage at 4 °C until biophysical analyses were conducted.

### Thermal melting assay

Thermal unfolding was monitored by light scattering and intrinsic fluorescence at 330 nm and 350 nm using a NanoTemper Prometheus instrument. Briefly, FIS1 or variants were prepared at a final concentration of 20 μM in 100 mM Hepes, pH 7.4, 200 mM NaCl, 1 mM DTT, 0.02% sodium azide. High-sensitivity capillaries (MO-K022) were then filled with each sample in four replicates for thermal scans. A melting scan was performed using an excitation power of 100%, a temperature range of 25 °C to 95 °C, and a temperature ramp of 0.5 °C/min. The resulting light scattering data were fit to a two-state model using the method of Santoro–Bolen equation (64) with the fit equation S(T) = ((S_F_ + m_F_∗T) + (S_U_ + m_U_∗T)∗exp(ΔH/R∗(1/T_m_-1/T)))/(1+exp(ΔH/R∗(1/T_m_-1/T))) to determine the midpoint of the unfolding transition, T_m_, and rendered as box and whisker plots using R (https://www.r-project.org/).

### NMR spectroscopy

Two-dimensional ^1^H,^15^ N heteronuclear single quantum coherence data were collected in 3 mm NMR tubes (Bruker) on a 14.1 T Bruker Avance II spectrometer equipped with a 5 mm TCI cryoprobe with a *z*-axis gradient. Data were collected on 100 μM ^15^N-FIS1 in 100 mM Hepes pH 7.4, 200 mM NaCl, 1 mM DTT, 0.02% (w/v) sodium azide, 10% ^2^H_2_O, 25 °C with eight transients, and 1024 (t2) × 300 (t1) complex points with acquisition times of 51.2 ms (^1^H) and 75 ms (^15^N). Spectra were processed with NMRPipe and analyzed with NMRAnalysis 2.5.2 (http://www.ccpn.ac.uk/software/analysis) (65) and NMRAssign 3.0 (https://ccpn.ac.uk/software/analysisassign/) (66) using NMRBox (67). Chemical shift assignments for FIS1 (1–125) have been previously reported (63) and for SKY variants were completed by visual inspection.

### Cell culture and transfections

HCT116 cells (American Type Culture Collection) were cultured in Mcoy5A supplemented with 10 mM glutamine, 10% fetal bovine serum (FBS), and 1% non-essential amino acid. See table of reagents in Supporting information for full details of chemicals and suppliers. Transfections were carried out in media supplemented with 2% FBS. For transfections, cells were plated on sterilized No. 1.5 glass bottom 24-well dishes (Cellvis). Optimal adherence and confluence were achieved by seeding cells at 20% confluence 48 h prior to transfection. Before transfection, cell media was changed to fresh media containing 2% FBS and 10 μM Quinoline-Val-Asp-Difluorophenoxymethyl Ketone. For transfections, plasmid DNA was added to Opti–minimal essential medium and briefly mixed by vortexing. The transfection reagent, Avalanche–Omni, was briefly vortexed and then 1 μl was added to the DNA:Opti–minimal essential medium mixture (1.25 μg:250 μl), immediately followed by vortexing for an additional 5 s. After 15 min of incubation at room temperature (RT), 100 μl of formed transfection complexes were added dropwise into each well. Cells were incubated in transfection reagent for 6 to 8 h, then changed to fresh media and incubated overnight. Cells were subsequently processed for immunofluorescence 18 to 24 h posttransfection.

### Immunofluorescence staining

Eighteen to twenty-four hours posttransfection, the medium was aspirated and replaced with 4% paraformaldehyde (prewarmed to 37 °C) and incubated with gentle shaking at RT for 25 to 30 min (see table of reagents in Supporting information for details). The fixative was removed and replaced with PBS. Following fixation, the cells were permeabilized by incubating with PBS/0.15% Triton X-100 for 15 min, followed by a brief wash in PBS and incubation with blocking solution (0.3% BSA/0.3% Triton X-100/PBS) for 1 h. Cells were then incubated overnight with primary antibody mix/5% normal goat serum/blocking solution, washed three times in PBS, incubated for 1 h with secondary antibody/blocking solution, and washed 2× in PBS/0.05% Tween 20 and once in PBS. To minimize antibody cross-reactivity in dual-labeling experiments, antibody incubations were processed sequentially, first for DRP1 (1:100) or Tom20 (1:500), followed by FIS1 (1:200).

### Image acquisition, FIS1-gating, colocalization, fluorescence intensity, and mitochondrial area analyses

Cells were visualized using a Nikon spinning-disk confocal microscope (see reagent table for detailed information). For morphology counts, cells were visualized using a 100× oil objective at 0.2-micron z-slices and 0.07-micron resolution and assessed by eye for the indicated morphology. Representative confocal images were acquired and processed using FIJI (https://imagej.net/software/fiji/). All immunofluorescence-based recruitment experiments were repeated three times and at least 30 cells or more (or a total of 100 or more cells) per experimental condition were manually cropped for statistical analyses. Prior to statistical analyses of morphology and DRP1 recruitment, FIS1-gating was done to exclude cells expressing higher than 1600 AU. For colocalization analysis, the FIJI coloc2 plugin was used to calculate Pearson’s correlation between endogenous DRP1 and mitoYFP, DRP1 and Tom20, or YFP-TBC1D15 and endogenous Tom20 as described (41). We note a limitation of this analysis is that it does not inform on the size differences in DRP1 or TBC1D15 punctate structures. A FIJI macro was used for cellular analyses and single-channel/single-cell z-stack images generated from MitoGraph preprocessing for the coloc2 analysis as described (41). Maximum intensity projection image stacks and images from MitoGraph preprocessing were used to measure the mean intensity of FIS1 within each cell. R was used to compile Pearson coefficients and combined in a merged dataset with the MitoGraph metrics and FIS1 fluorescence intensity analysis as described (41). For analyses of YFP-TBC1D15 signal transition, YFP fluorescence intensity analyses were similarly performed in batch mode on FIJI using MitoGraph preprocessing cropped images to determine YFP mean and mode values per cell. Violin plots and ANOVA statistical calculations were also performed using R.

Batch mode preprocessing of images for mitochondrial area assessment by MitoGraph was done using R scripts previously described (41, 68). MitoGraph segmentation and noise removal were performed on cropped Tag Image File Format files using the following commands for segmentation: MitoGraph -xy 0.07 -z 0.2 -adaptive 10 -path cells. The resulting Portable Network Graphic files were compiled using an ImageJ (https://imagej.net/ij/) macro and screened for accurate mitochondrial segmentation as previously described (41). The average mitochondrial area was then determined by multiplying the average edge length and average width values generated by MitoGraph. Mitochondrial area data was merged with mean fluorescence intensity values of FIS1 for statistical evaluation using R.

### Western blot

Transfected HCT116 cells were harvested using a radioimmunoprecipitation assay lysis kit (ProteinSimple CBS401), and cleared supernatants were saved at −20 °C until analyses. Capillary electrophoresis experiments were carried out using a JESS system (ProteinSimple) with the 25 capillary 12 to 230 kDa Separation module (ProteinSimple SM-W004), FIS1 antibody (Proteintech 10955-1-AP), and the Anti-Rabbit Detection Module (ProteinSimple DM-001). Setup and analysis were performed according to the manufacturer’s instructions. Briefly, samples were diluted to a final concentration of 0.2 mg/ml in 0.1× sample buffer and 5× fluorescent master mix. The biotinylated ladder and the samples are then heated at 95 °C for 5 min. Once all reagents were dispensed, the plate was covered, and centrifuged for 5 min at 1000 rpm. Runs were performed using the instrument default settings in the Compass software (ProteinSimple, version 6.1.0; https://www.bio-techne.com/resources/instrument-software-download-center/compass-software-simple-western). Once the run is complete, we use the Compass software to determine the signal area for each antibody. For area calculations, we use the dropped lines option. We additionally performed a total protein assay for loading level normalization using the Total Protein Detection Module (DM-TP01). The total protein area for FIS1 was normalized to overexpressed wildtype FIS1 and plotted for comparison. Conventional Western blots were developed and imaged by enhanced chemiluminescence (Bio-Rad) and quantified by densitometry using the gel analyses tool on FIJI (ImageJ).

### Coimmunoprecipitation

Briefly, transfected HCT116 cells were harvested by trypsinization, washed with 1× Hanks' balanced salt solution (HBSS), and transferred to prechilled tubes. Washed cell pellets were cross-linked by resuspending in cross-linking buffer (0.25% paraformaldehyde in 1× HBSS) and incubating either on ice or at RT for 8 min. The reaction was subsequently quenched by adding an equal volume of quenching buffer (1.25 M glycine in 1× HBSS). The recovered cell pellets were then resuspended in 300 μL of lysis buffer (10 mM Tris/Cl pH 7.5, 150 mM NaCl, 0.5 mM EDTA, 0.5 % Nonidet P40 Substitute). Resuspended whole-cell lysates were then pulse sonicated on ice and incubated for at least 1 h at 4 °C to completely recover cross-linked complexes. At 4 °C, whole-cell lysates were centrifuged at 14,000 RPM for 10 min, and the supernatants were carefully collected in fresh prechilled tubes. Lysates were precleared with 10 μl of protein A/G beads, 30 μl was collected for input fractions, and the remaining supernatant was used for coimmunoprecipitation. Endogenous DRP1 was immunoprecipitated using a mouse monoclonal DRP1 antibody (sc-271583) pre-conjugated to A/G beads. Ectopic YFP-TBC1D15 was immunoprecipitated using a GFP-nanobody (GFP-trap Agarose, Proteintech GTA20). Coimmunoprecipitates were recovered for SDS-PAGE and Western blot by boiling beads in 2.5× Laemmli buffer.

## Data availability

All R scripts used for data analysis and visualization are available upon request and/or for download at https://github.com/Hill-Lab/. Raw data is available upon request.

## Supporting information

This article contains supporting information.

## Conflict of interest

R. B. H. and R. T. have financial interest in Cytegen, a company developing therapies to improve mitochondrial function. A portion of the salary for R. T. is paid for by the company. Cytegen had no role in study design; in the data collection, analysis and interpretation of data; in the writing of the report; and in the decision to submit the article for publication. The other authors declare that they have no conflicts of interest with the contents of this article.
