# Supplementary material for: A conserved, noncanonical insert in FIS1 mediates TBC1D15 and DRP1 recruitment for mitochondrial fission
Source: J Biol Chem. 2023 Sep 28;299(11):105303. doi: 10.1016/j.jbc.2023.105303 (PMC10641528; doi:10.1016/j.jbc.2023.105303)
Supplement: Supplementary figures [file mmc1.pdf]

Supporting Information for Ihenacho et al

"A conserved, non-canonical insert in FIS1 mediates TBC1D15 and DRP1 recruitment for mitochondrial fission"

Figure S1. Rational design and validation of ΔSKY FIS1 variants

S1, ^{1}H-^{15}N HSQC spectral overlays of FIS1 wildtype (black) with ΔSKYD49G(left panel) and ΔNΔSKYD49G (right panel) with colors as indicated. Data were collected on 100 µM samples at 25 °C, pH 7.4 at 14.1 T. FIS1 arm crosspeaks are indicated in magenta. **S1A,** The midpoint of the thermal unfolding transition was determined by fitting light scattering data collected from 25-95°C with the mean ± standard deviation from 3-5 technical replicates shown as a box-and-whisker plot. **S1B-F,** Full ^{1}H-^{15}N HSQC spectral overlays of FIS1 wildtype (black) with ΔNΔSKYD49G, AAA, ΔSKY, ΔSKYD49G, and D49G, respectively. Arm residue crosspeaks are labeled in magenta.

Figures S2-S3. Confocal image gating methods and correlational analyses.

S2A, Western blot showing FIS1 expression in HCT116 cells co-transfected with pcDNA-mitoYFP and pcDNA-FIS1. FIS1 expression in each sample is first normalized to total protein expression and then quantified (n=1) as relative ratios to wildtype FIS1. **S2B,** Prior to analyzing confocal microscopy images shown in **Figure 3**, cells were gated to exclude cells that overexpressed FIS1 (mean A.U. >1600). Each point in the scatter plot represents a cell cropped for image analysis. Only cells with A.U. < 1600 were included in quantitative analyses presented in Figure 3. **S2C,** Correlational plots to determine the relationship between FIS1 expression and mitochondrial network area, and DRP1 recruitment (**S2D**). Each point is colored based on the gated population average FIS1 expression of gated cells.

S3A, Scatter plots of all cropped cells from confocal microscopy images shown in **Figure 5,** showing the relationship between FIS1 expression and mitochondrial network area and DRP1 recruitment. (**S3B**) without and without ectopic YFP-TBC1D15. The red shaded area in **S3A** and **S3B** shows which cells were included for analyses after gating. **S3C,** Correlational plots to determine the relationship between FIS1 expression and mitochondrial network area and DRP1 recruitment (**S3D**). Each point is colored based on the gated population's average FIS1 expression of gated cells. **S3E**, Western blot showing FIS1 and YFP-TBC1D15 expression levels (top), and the quantification (n =1) of relative expression to wildtype FIS1 (bottom).

Fig. S1.

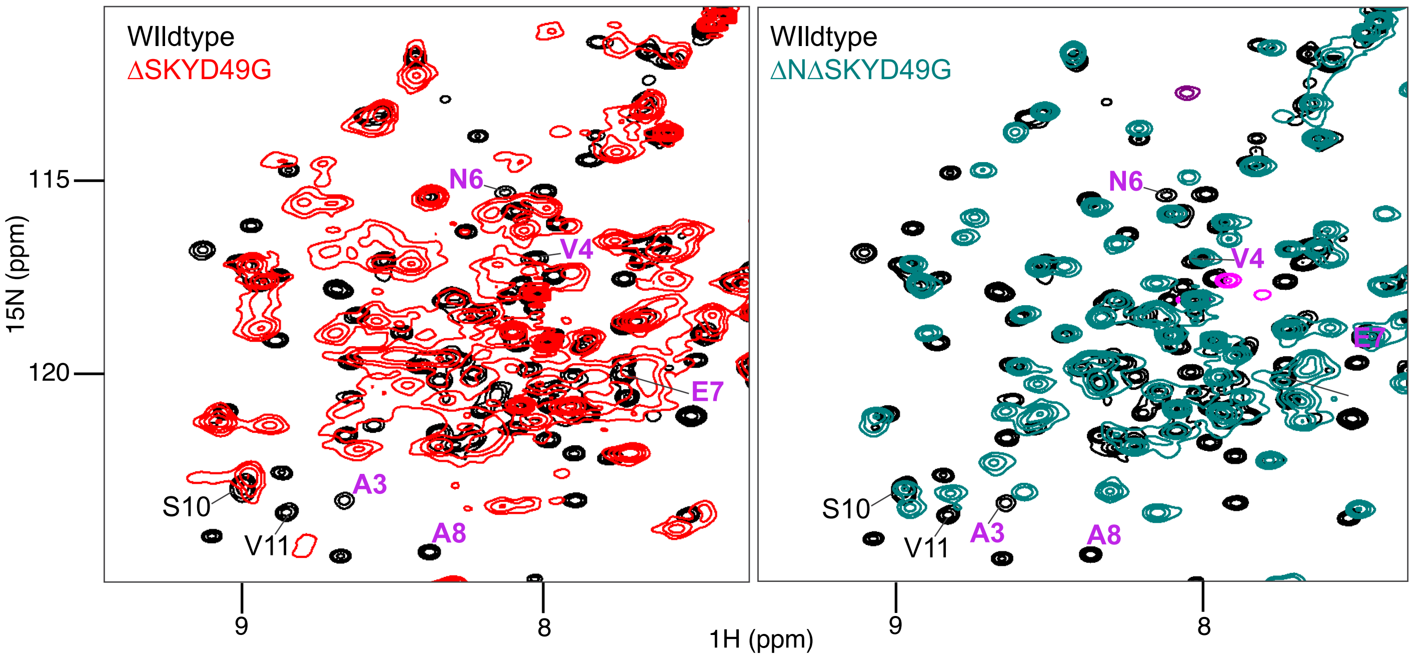

Fig. S1a.

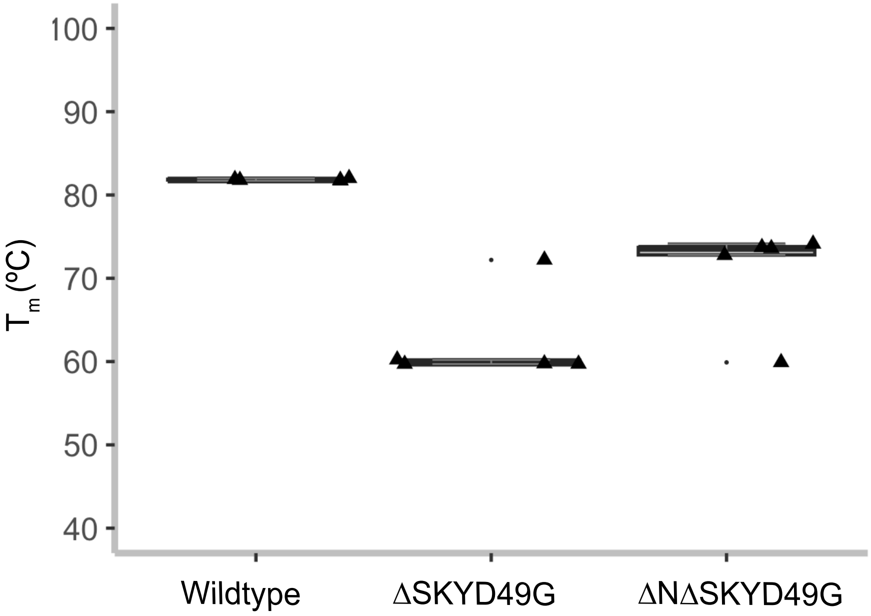

S1b.

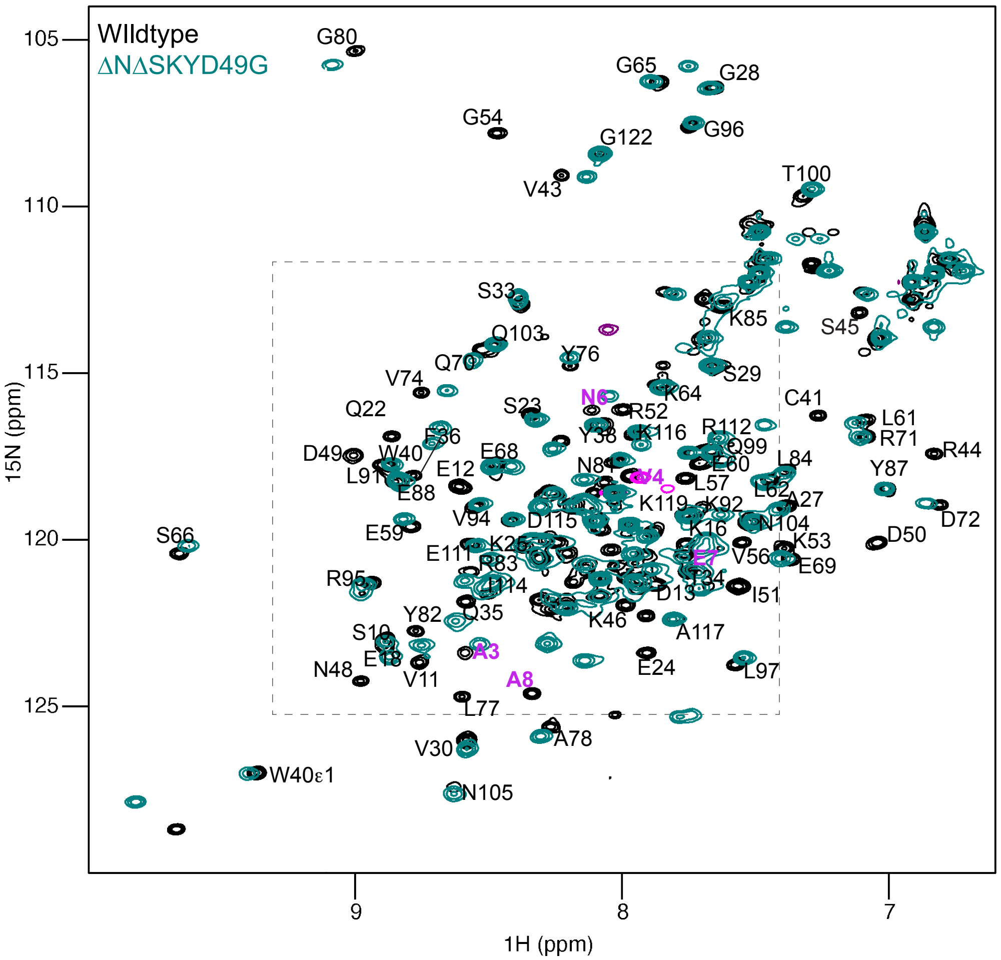

WIldtype
ΔNΔSKYD49G
15N (ppm)
1H (ppm)
105
110
115
120
125
9
8
7
G80
G65
G28
G54
G122
G96
V43
T100
S33
K85
S45
Q103
Y76
Q70
S29
V74
K64
C41
Q22
S23
N6
R52
L61
R71
F36
Y38
K116
R112
Q99
R44
D49
W40
E68
E60
L84
Y87
L91
E12
N8
V4
L57
E88
L62
A27
V94
D115
K119
K92
D72
E59
N104
D50
K16
K53
E111
K25
E7
V56
E69
R83
R95
I114
T34
D13
I51
Y82
Q35
K46
A117
S10
A3
E18
N48
V11
A8
E24
L97
L77
A78
V30
W40ε1
N105
S66

S1c.

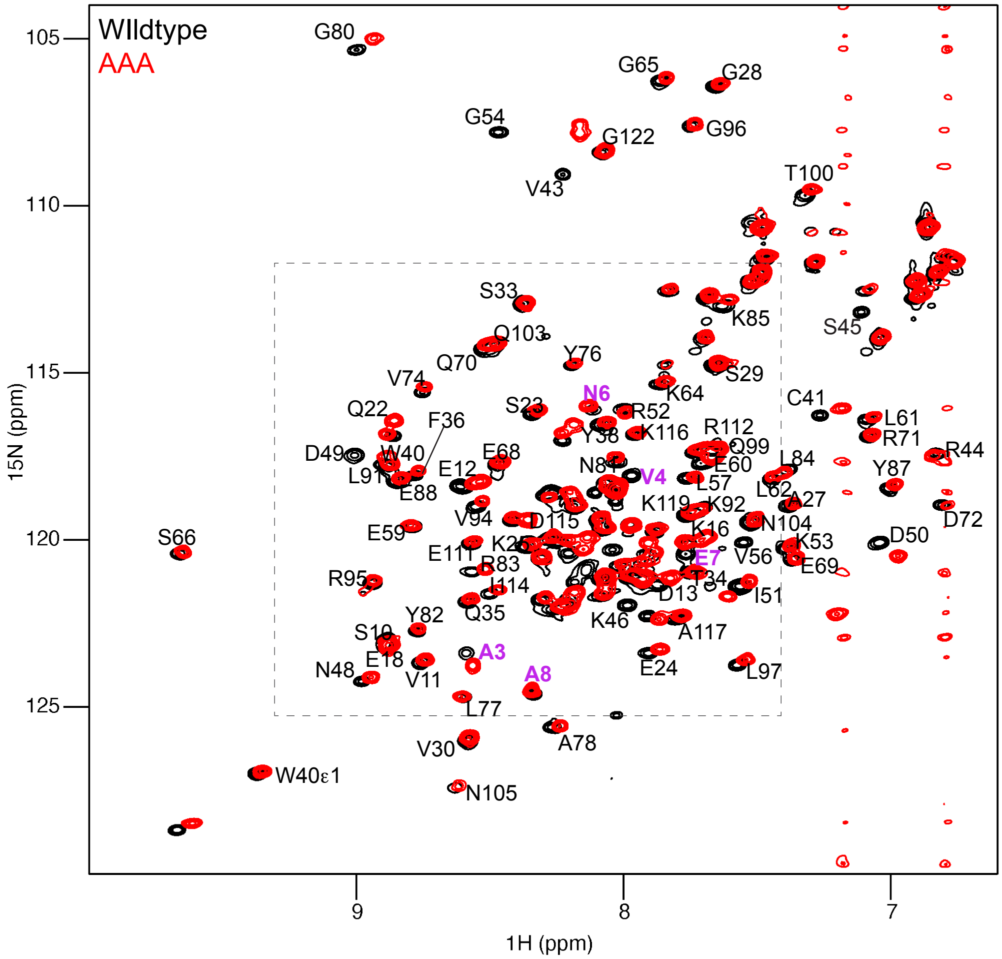

WIldtype
AAA
15N (ppm)
1H (ppm)
105
110
115
120
125
9
8
7
G80
G65
G28
G54
G122
G96
V43
T100
S33
K85
S45
Q103
Y76
Q70
S29
V74
K64
C41
Q22
S23
N6
R52
L61
R71
F36
Y38
K116
R112
Q99
R44
D49
W40
E68
E60
L84
Y87
L91
E12
N8
V4
L57
E88
L62
A27
V94
D115
K119
K92
D72
E59
N104
D50
K16
K53
E111
K25
E7
V56
E69
R83
R95
I114
T34
D13
I51
Y82
Q35
K46
A117
S10
A3
E18
N48
V11
A8
E24
L97
L77
A78
V30
W40ε1
N105
S66

S1d.

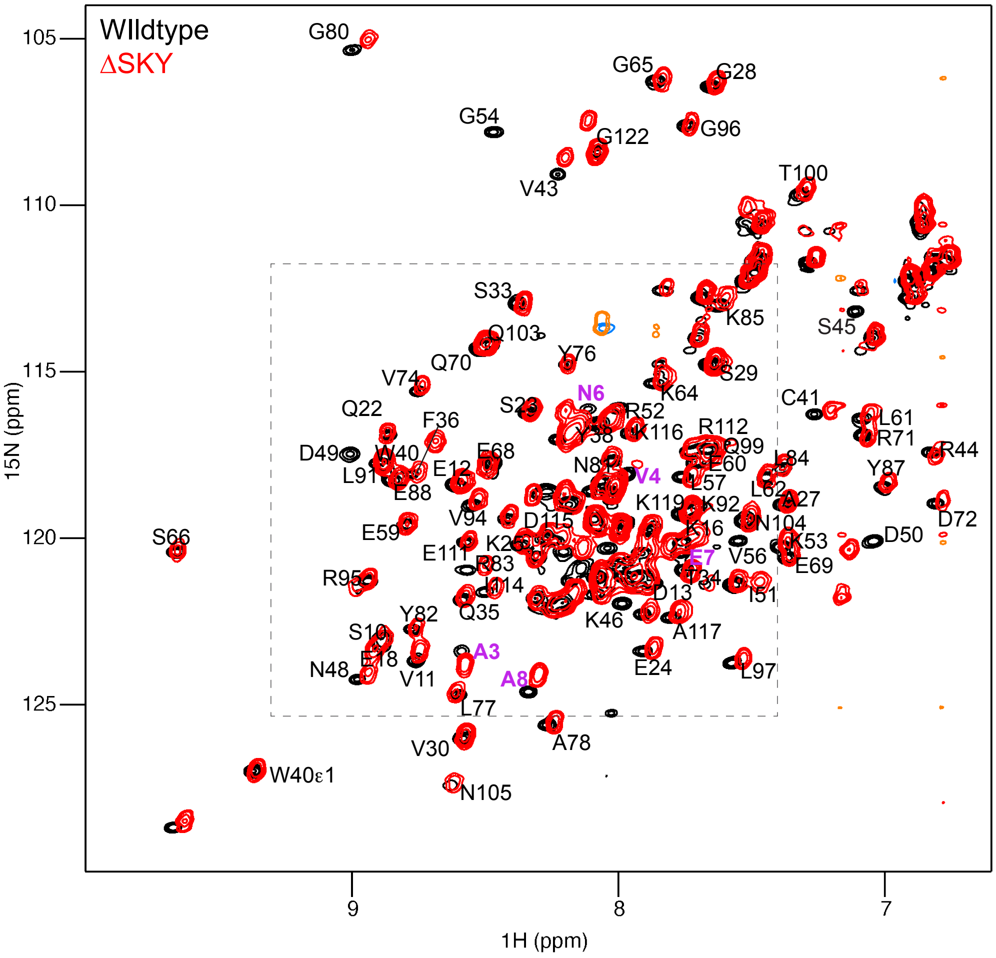
WIldtype
ΔSKY
G80
G65
G28
G54
G122
G96
V43
T100
S33
K85
Q103
S45
Y76
Q70
S29
V74
N6
K64
C41
Q22
S23
R52
L61
F36
Y38
K116
R112
R71
D49
W40
F68
Q99
R44
L91
E12
N87
E60
L84
Y87
E88
V4
L57
L62
A27
K119
K92
V94
D115
N104
D72
E59
E16
K53
D50
E111
K26
V56
E7
R83
E69
R95
I114
T34
I51
D13
Y82
Q35
K46
A117
S10
A3
E18
E24
N48
V11
A8
L97
L77
A78
V30
W40ε1
N105
S66
105
110
115
120
125
15N (ppm)
9
8
7
1H (ppm)

S1e.

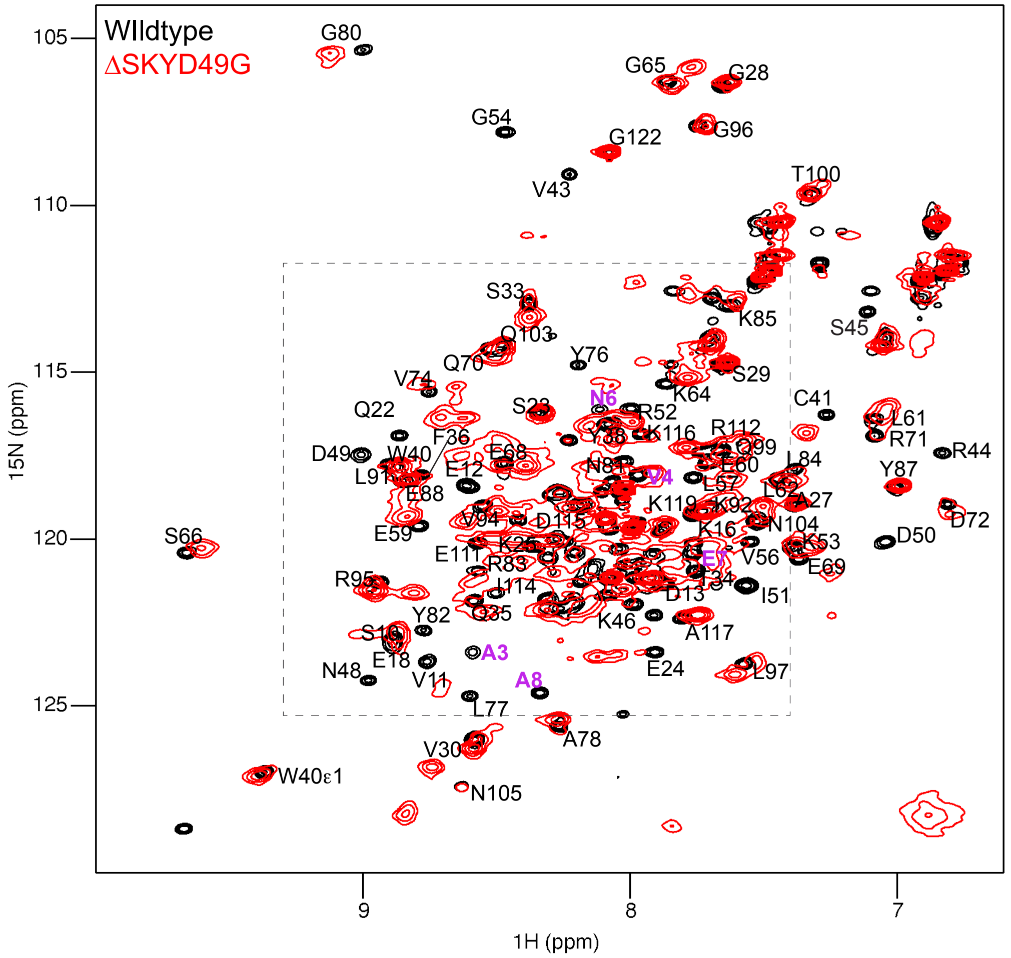
WIldtype
ΔSKYD49G
G80
G65
G28
G54
G122
G96
V43
T100
S33
K85
Q103
S45
Y76
Q70
S29
V74
N6
K64
C41
Q22
S23
R52
L61
F36
Y38
K116
R112
R71
D49
W40
F68
Q99
R44
L91
E12
N87
E60
L84
Y87
E88
V4
L57
L62
A27
K119
K92
V94
D115
N104
D72
E59
E16
K53
D50
E111
K26
V56
E7
R83
E69
R95
I114
T34
I51
D13
Y82
Q35
K46
A117
S10
A3
E18
E24
N48
V11
A8
L97
L77
A78
V30
W40ε1
N105
S66
105
110
115
120
125
15N (ppm)
9
8
7
1H (ppm)

S1f.

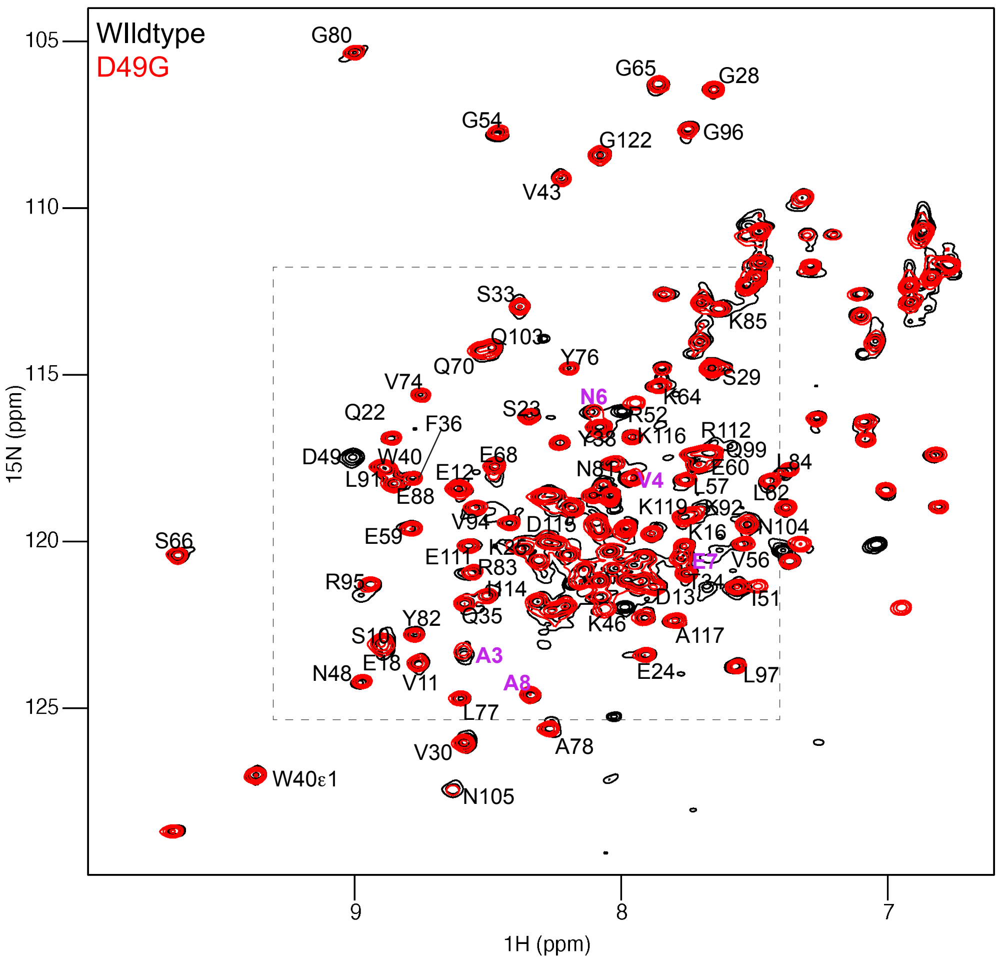
WIldtype
D49G
105
110
115
120
125
15N (ppm)
9
8
7
1H (ppm)
G80
G65
G28
G54
G122
G96
V43
S33
Q103
Y76
Q70
V74
S29
K85
K64
N6
Q22
F36
S23
R52
Y38
K116
R112
Q99
D49
W40
E68
E12
N81
E60
L84
L91
E88
V4
L57
L62
K119
K92
V94
D119
N104
E59
K26
K16
E111
R83
E7
V56
S66
R95
H14
I51
D13
Y82
Q35
K46
A117
S19
A3
E18
E24
L97
N48
V11
A8
L77
A78
V30
W40ε1
N105

Fig. S2A.

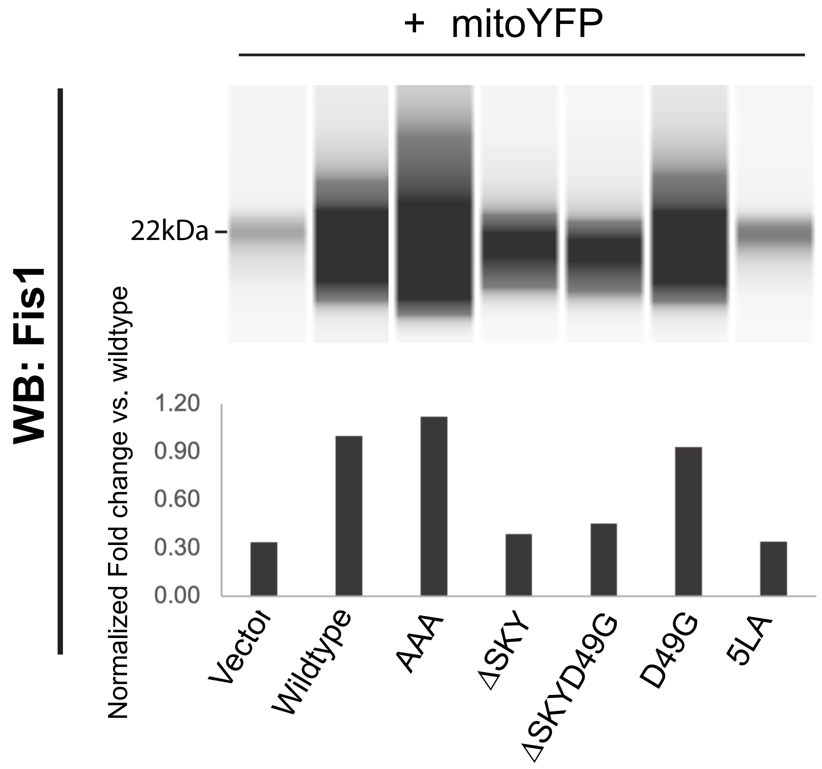
+ mitoYFP
WB: Fis1
22kDa
Normalized Fold change vs. wildtype
1.20
0.90
0.60
0.30
0.00
Vector
Wildtype
AAA
ΔSKY
ΔSKYD49G
D49G
5LA

Fig. S2A. continued

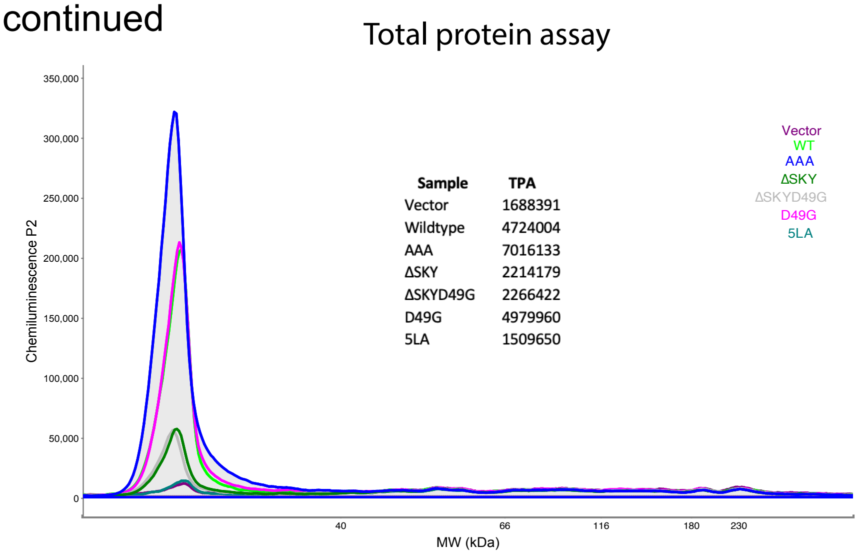

Fig. S2B.

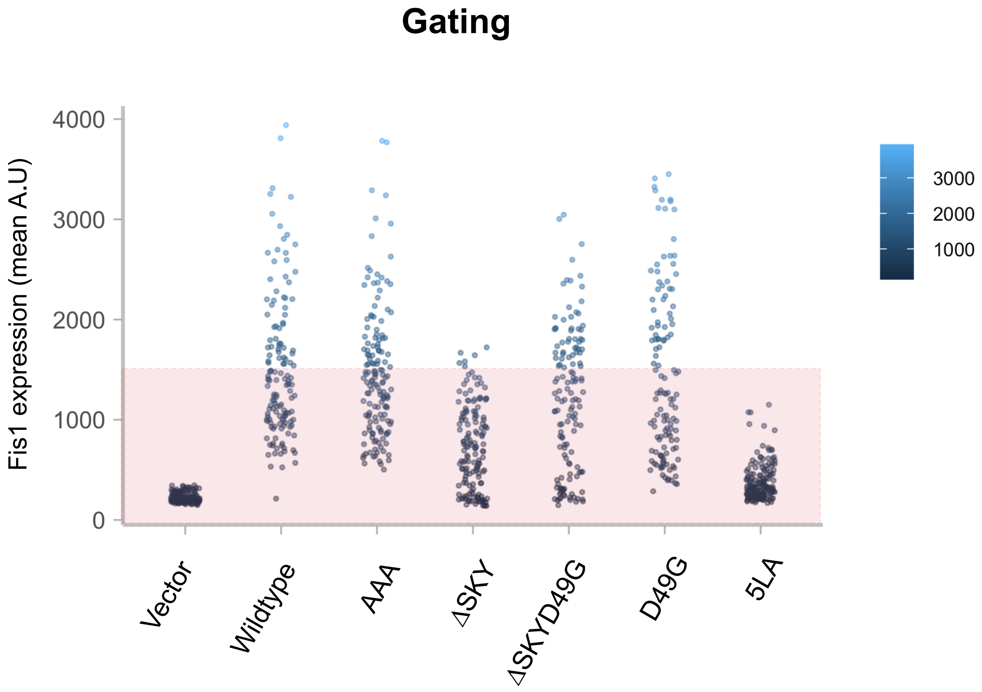

S2C.

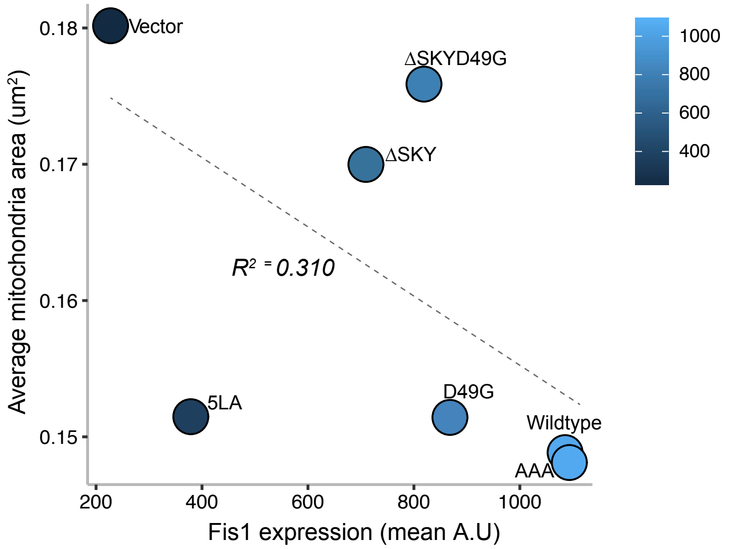

S2D.

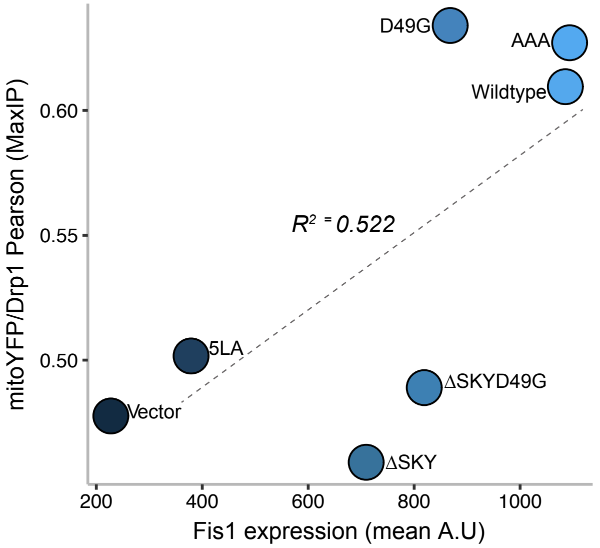

Fig. S3A.

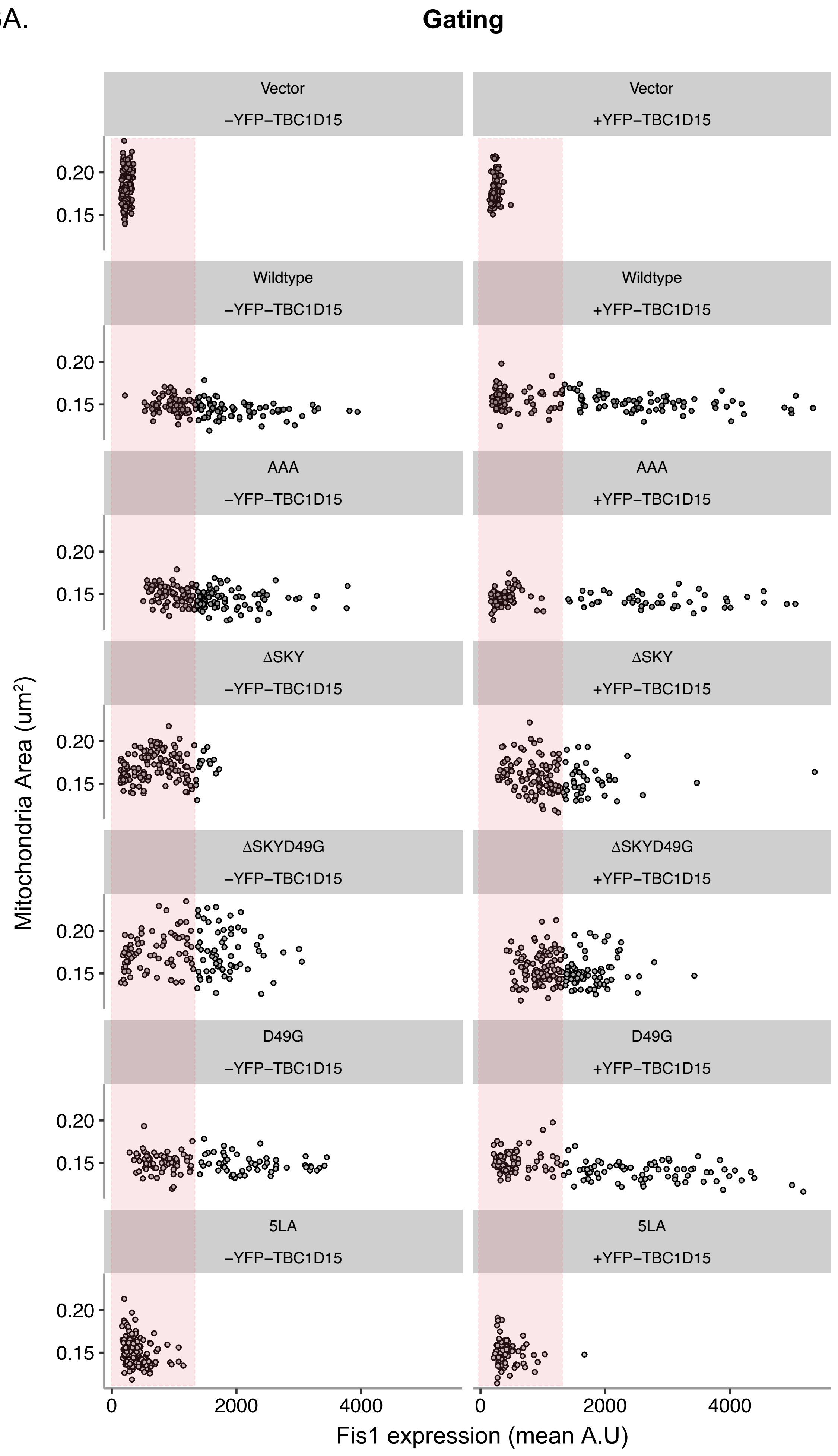
Gating
Vector
−YFP−TBC1D15
Vector
+YFP−TBC1D15
Wildtype
−YFP−TBC1D15
Wildtype
+YFP−TBC1D15
AAA
−YFP−TBC1D15
AAA
+YFP−TBC1D15
ΔSKY
−YFP−TBC1D15
ΔSKY
+YFP−TBC1D15
ΔSKYD49G
−YFP−TBC1D15
ΔSKYD49G
+YFP−TBC1D15
D49G
−YFP−TBC1D15
D49G
+YFP−TBC1D15
5LA
−YFP−TBC1D15
5LA
+YFP−TBC1D15
0.20
0.15
Mitochondria Area (um²)
0
2000
4000
Fis1 expression (mean A.U)

Fig. S3B.

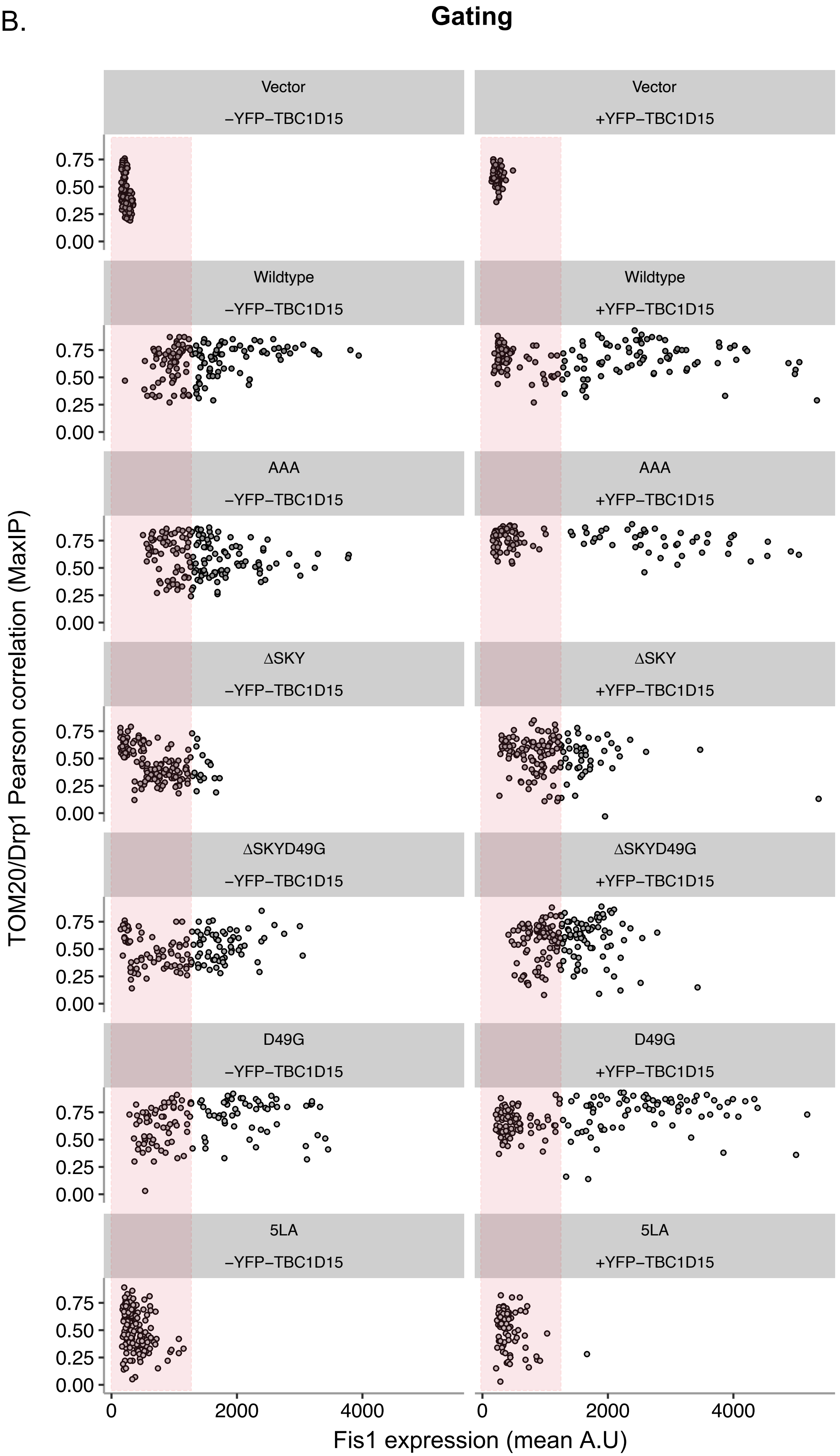
Gating
Vector
−YFP−TBC1D15
Vector
+YFP−TBC1D15
Wildtype
−YFP−TBC1D15
Wildtype
+YFP−TBC1D15
AAA
−YFP−TBC1D15
AAA
+YFP−TBC1D15
ΔSKY
−YFP−TBC1D15
ΔSKY
+YFP−TBC1D15
ΔSKYD49G
−YFP−TBC1D15
ΔSKYD49G
+YFP−TBC1D15
D49G
−YFP−TBC1D15
D49G
+YFP−TBC1D15
5LA
−YFP−TBC1D15
5LA
+YFP−TBC1D15
0.00
0.25
0.50
0.75
0
2000
4000
TOM20/Drp1 Pearson correlation (MaxIP)
Fis1 expression (mean A.U)

Fig. S3C.

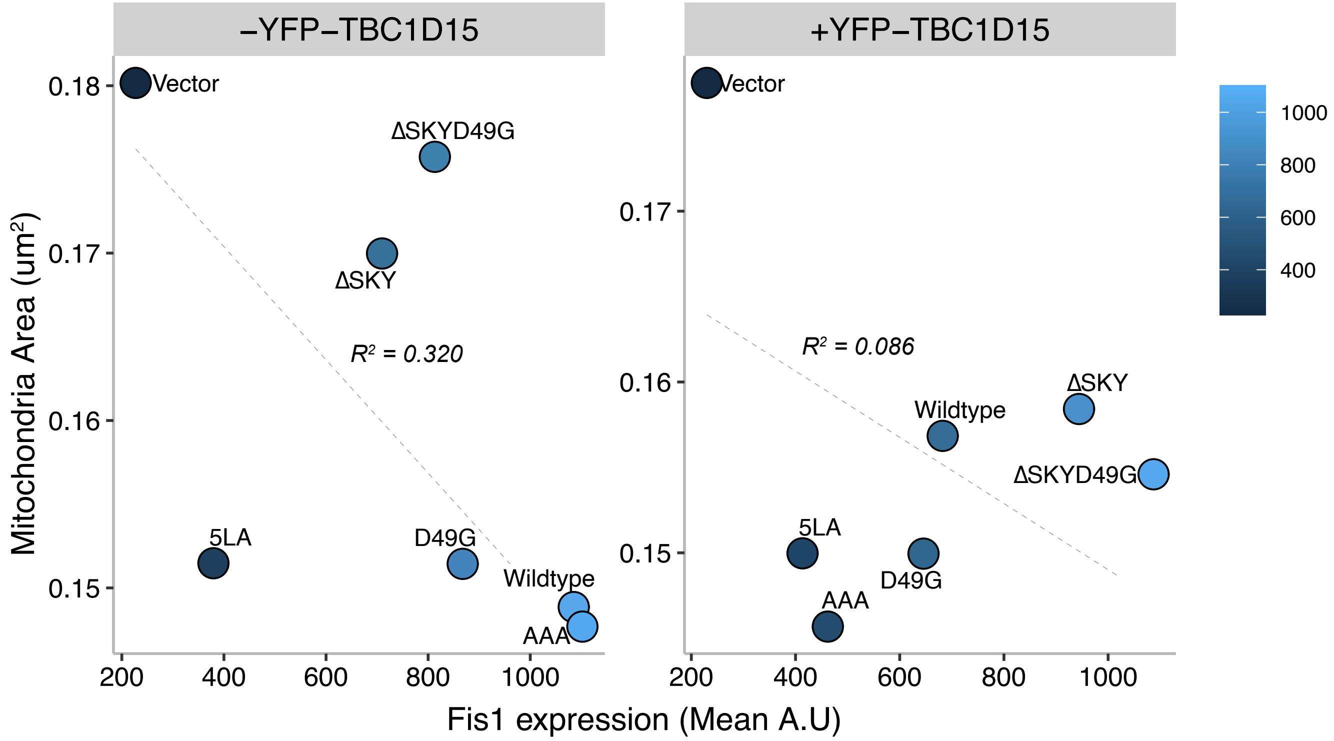
−YFP−TBC1D15
+YFP−TBC1D15
Mitochondria Area (um²)
Fis1 expression (Mean A.U)
0.18
0.17
0.16
0.15
200
400
600
800
1000
Vector
ΔSKYD49G
ΔSKY
R² = 0.320
5LA
D49G
Wildtype
AAA
R² = 0.086
ΔSKY
Wildtype
ΔSKYD49G
5LA
D49G
AAA
1000
800
600
400

S3D.

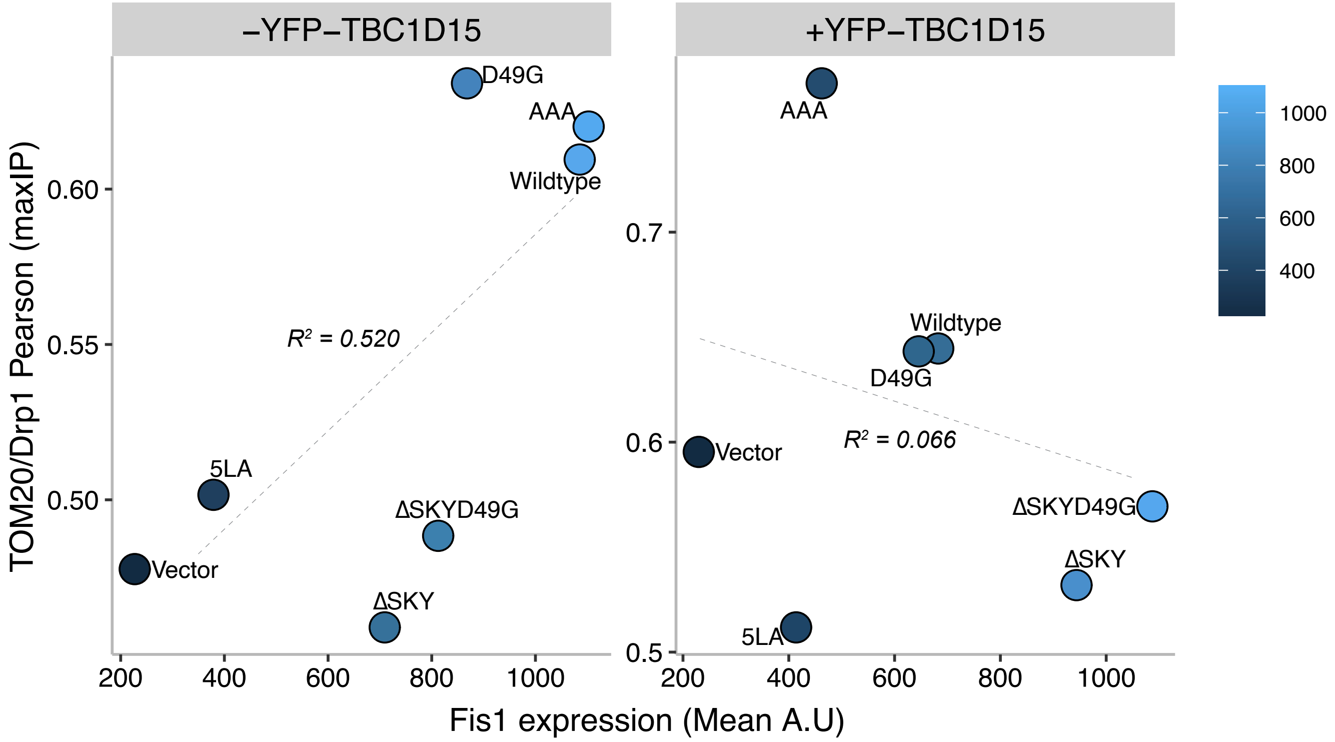
−YFP−TBC1D15
+YFP−TBC1D15
TOM20/Drp1 Pearson (maxIP)
Fis1 expression (Mean A.U)
0.60
0.55
0.50
0.7
0.6
0.5
200
400
600
800
1000
D49G
AAA
Wildtype
R² = 0.520
5LA
ΔSKYD49G
Vector
ΔSKY
AAA
Wildtype
D49G
Vector
R² = 0.066
ΔSKYD49G
ΔSKY
5LA
1000
800
600
400

S3E.

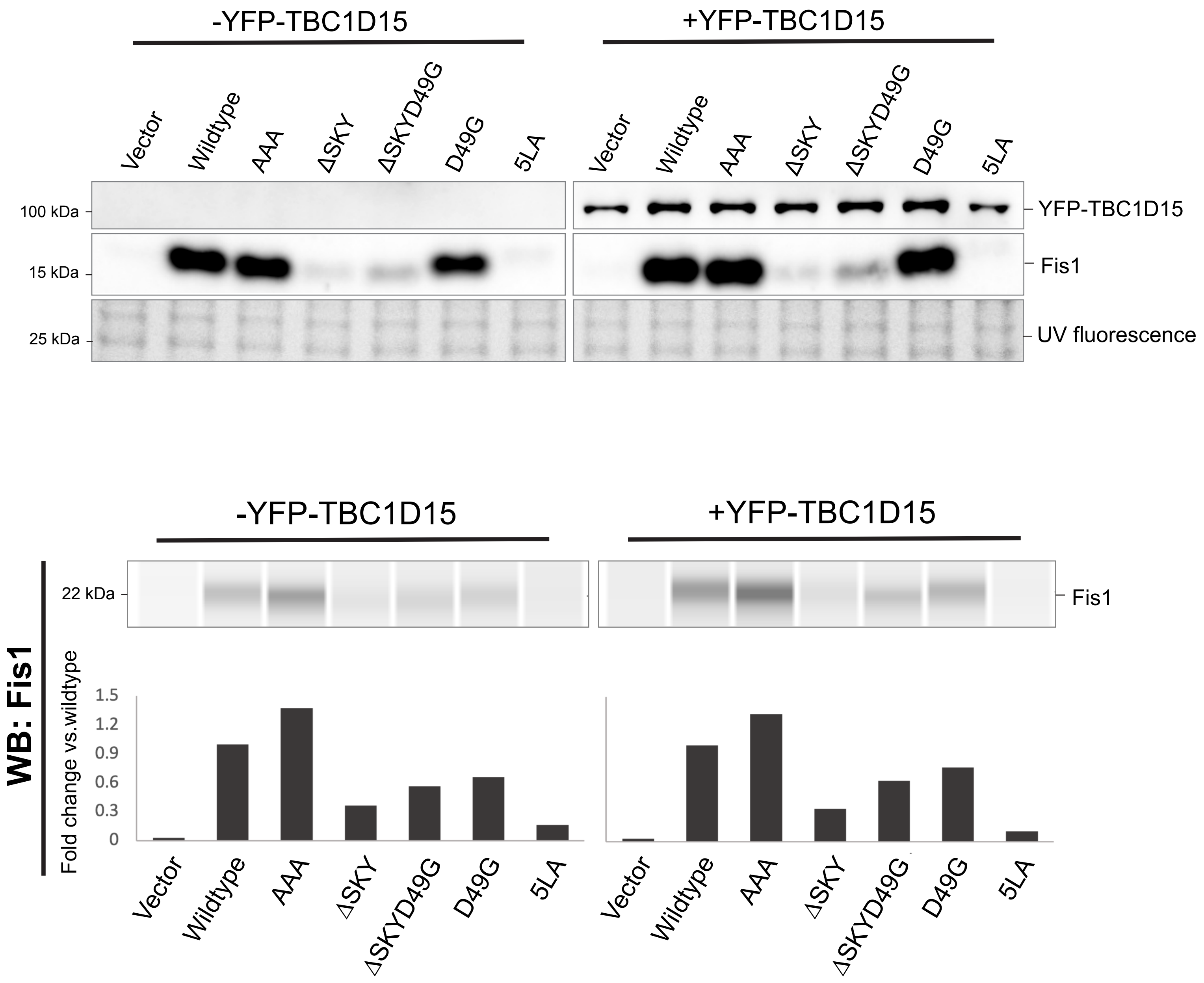
-YFP-TBC1D15
+YFP-TBC1D15
Vector
Wildtype
AAA
ΔSKY
ΔSKYD49G
D49G
5LA
100 kDa
15 kDa
25 kDa
YFP-TBC1D15
Fis1
UV fluorescence
22 kDa
WB: Fis1
Fold change vs.wildtype
1.5
1.2
0.9
0.6
0.3
0
