# Supplementary material for: A conserved, noncanonical insert in FIS1 mediates TBC1D15 and DRP1 recruitment for mitochondrial fission
Source: J Biol Chem. 2023 Sep 28;299(11):105303. doi: 10.1016/j.jbc.2023.105303 (PMC10641528; doi:10.1016/j.jbc.2023.105303)
Supplement: Supporting information [file mmc2.docx]

Supporting Information for Ihenacho et al

“**A conserved, non-canonical insert in FIS1 mediates TBC1D15 and DRP1 recruitment for mitochondrial fission”**

**Figure S1. Rational design and validation of ΔSKY FIS1 variants**

**S1,** ^1^H-^15^N HSQC spectral overlays of FIS1 wildtype (black) with ∆SKYD49G(left panel) and ∆N∆SKYD49G (right panel). Data were collected on 100 µM samples at 25 °C, pH 7.4 at 14.1 T.  FIS1 arm crosspeaks are indicated in magenta. **S1A,** The midpoint of the thermal unfolding transition was determined by fitting light scattering data collected from 25-95°C with the mean ± standard deviation from 3-5 technical replicates shown as a box-and-whisker plot. **S1B-F,** Full ^1^H-^15^N HSQC spectral overlays of FIS1 wildtype (black) with ∆N∆SKYD49G, AAA, ∆SKY, ∆SKYD49G, and D49G, respectively. Arm residue crosspeaks are labeled in magenta.

**Figures S2-S3. Confocal image gating methods and correlational analyses.**

**S2A,** Western blot showing FIS1 expression in HCT116 cells co-transfected with pcDNA-mitoYFP and pcDNA-FIS1. FIS1 expression in each sample is first normalized to total protein expression and then quantified (n=1) as relative ratios to wildtype FIS1.  **S2B,** Prior to analyzing confocal microscopy images shown in **Figure 3**, cells were gated to exclude cells that overexpressed FIS1 (mean A.U >1600). Each point in the scatter plot represents a cropped cell **S2C,** Correlational plots to determine the relationship between FIS1 expression and mitochondrial network area, and DRP1 recruitment (**S2D**). Each point is colored based on the gated population average FIS1 expression of gated cells.

**S3A,** Scatter plots of all cropped cells from confocal microscopy images shown in **Figure 5,** showing the relationship between FIS1 expression and mitochondrial network area and DRP1 recruitment. (**S3B**) without and without ectopic YFP-TBC1D15. The red shaded area shows which cells were included for analyses after gating. **S3C,** Correlational plots to determine the relationship between FIS1 expression and mitochondrial network area and DRP1 recruitment (**S3D**). Each point is colored based on the gated population’s average FIS1 expression of gated cells. **S3E**, Western blot showing FIS1 and YFP-TBC1D15 expression levels (top), and the quantification (n =1) of relative expression to wildtype FIS1 (bottom).
